# A Physically Active Status Affects the Circulating Profile of Cancer-Associated miRNAs

**DOI:** 10.3390/diagnostics11050820

**Published:** 2021-04-30

**Authors:** Martina Faraldi, Laura Gerosa, Marta Gomarasca, Veronica Sansoni, Silvia Perego, Ewa Ziemann, Giuseppe Banfi, Giovanni Lombardi

**Affiliations:** 1 Laboratory of Experimental Biochemistry & Molecular Biology, IRCCS Istituto Ortopedico Galeazzi, 20161 Milano, Italy; martina.faraldi@grupposandonato.it (M.F.); marta.gomarasca@grupposandonato.it (M.G.); veronica.sansoni@grupposandonato.it (V.S.); silvia.perego@grupposandonato.it (S.P.); banfi.giuseppe@unisr.it (G.B.); giovanni.lombardi@grupposandonato.it (G.L.); 2 Gruppo San Donato Foundation, 20122 Milano, Italy; 3 Department of Athletics, Strength and Conditioning, Poznań University of Physical Education, 61-871 Poznań, Poland; ziemann@awf.poznan.pl; 4 Vita-Salute San Raffaele University, 20132 Milano, Italy

**Keywords:** circulating miRNAs, cancer biomarkers, physical activity, pre-analytical variables

## Abstract

Circulating miRNAs are ideal diagnostics and prognostics biomarkers in cancer since altered levels of specific miRNAs have been associated to development/progression of several cancers. Physical activity is a recognized preventive strategy against several cancers, but it may also modify the baseline levels of cancer-associated miRNAs and, hence, may act as a confounding pre-analytical variable. This study aimed at understanding whether physical activity-dependent changes in cancer-associated circulating miRNAs profile could act as a confounding variable. A panel comprising 179 miRNAs was assayed in plasma from 20 highly trained and 10 sedentary men. RT-qPCR data were analyzed with the 2^−2ΔΔCT^ methods and normalized on hsa-miR-320d, as determined by bioinformatics analysis. miRNAs associated with the diagnosis of the most prevalent cancers were considered. Only those miRNAs, relevantly associated with cancers, found ≥2-fold up- or downregulated in highly trained subjects compared to sedentary were disclosed. The results reveal that chronic physical activity determined modifications altering the baseline level of several cancer-associated miRNAs and, hence, their diagnostic and prognostic potential. In conclusion, based on our results, a physically active status emerges as an important pre-analytical variable able to alter the basal level of circulating miRNAs, and these alterations might be considered as potentially misleading the analytical output.

## 1. Introduction

MicroRNAs (miRNAs) are small non-coding RNA molecules (18–22 nucleotides in length), actively regulating gene expression [1,2]. Several miRNAs display a certain degree of cell- or tissue-specificity, while others are more broadly expressed [3]. Since released in biological fluids [4], they can be detected and quantified in blood [5]. Although circulating miRNAs levels are very low and, however, much lower than in the tissue/cells source [6], they give particular advantage in the case of tissues hardly subjectable to bioptic procedures (e.g., bone and cartilage) [7,8] and/or to serial monitoring the progress of diseases, such as osteoporosis, fracture risk [9] and tumors [10], as well as the response to physical activity [11,12].

During neoplastic transformation, miRNAs may be aberrantly expressed and miRNA signatures can be associated with specific tumors and, possibly, with a given tumor stage [13]. Altered circulating miRNA profiles have been associated with transformation and tumor growth, progression, metastasis and development of drug resistance. Consequently, circulating miRNAs profiling is of great interest in cancer diagnosis and prognosis [14]. Further, circulating miRNAs do not only passively mark a pathological condition, by mirroring the tumor cell content, but they can actively act as paracrine/endocrine mediators that target other tissues to determine, for example, the appropriate microenvironment to host a metastasis [15].

Physical inactivity (PI) is the fourth leading cause of death (5.5% of deaths globally) after hypertension, cigarette smoking and hyperglycemia and just before obesity and overweight [16]. PI independently associates with an increased incidence of cancers in general and specifically for certain tumors (e.g., colorectal, lung, breast, prostate, ovarian, endometrial and esophageal) [17]. The net energy surplus consequent to the sedentary behavior is the underlying cause of the onset of these diseases [18]. The preventive role of exercise training, and particularly of endurance training, is explicated towards a plethora of not completely understood molecular mechanisms [19]. Among these, acute and chronic physical activity (PA) profoundly affects the circulating miRNA profile [20]. The anti-cancer effect of PA may thus be mediated, at least in part, by the alteration of miRNA expression profile: PA may determine a miRNA-based regulatory network that make neoplastic transformation, tumor progression and metastasis less probable [21]. From a diagnostic point of view, however, the physiological differences between sedentary and trained subjects must be considered in a clinical setting since they represent an important source of variability. As such, PA is a pre-analytical variable to be accounted in routine analysis especially when highly sensitive analytes are assayed, such as miRNAs [12,22], but thus far no published papers have investigated how PA affects the circulating miRNA-based diagnosis/prognosis on cancer [21]. Based on this background, this study aimed at determining to what extent a physically active status may act as a pre-analytical variable potentially modifying the diagnostic value of miRNAs in cancer, by comparing the circulating miRNA profile of highly trained subjects and sedentary individuals.

## 2. Materials and Methods

### 2.1. Study Population

We recruited 24 volunteer young adult males, preselected based on their physical activity profile. Eligibility criteria were: age range 25–40 years, healthy status, normal body mass index (BMI, 20 ≥ BMI ≥ 25 kg/m^2^), non-smokers, no recent (in the preceding month) or chronic diseases and/or medications. According to the guidelines from the American College of Sports Medicine (ACSM) [23], participants were grouped as either sedentary or endurance trained. Sedentary individuals (*n* = 10) perform less than 30 min, 5 days/week (or 150 min/week) of moderate intensity PA. In our setting, endurance trained individuals (*n* = 14) were non-professional mountain ultra-trail athletes accustomed to vigorous-to-high intensity, high volume, long-lasting endurance PA [23] that were recruited at the end of the 9th week of preparation (tapering week, during which the training volumes are halved [24]) for an ultra-trail mountain marathon. All individuals gave their written consent after being informed about study aim, protocol, procedures, associated benefits and eventual risks, in accordance with the Helsinki Declaration (SportMarker protocol approved by the Comitato Etico Ospedale San Raffaele, Milano, Italy and registered on ClinicalTrials.gov NCT03386981).

### 2.2. Blood Sampling

All participants were asked to abstain from alcohol, keep a regular diet and avoid any pro-inflammatory activity (shaving, waxing, etc.) in the week before the sampling. Sedentary participants were asked to avoid any PA in the 48 h preceding blood sampling. Trained subjects, instead, were sampled the day before an ultra-trail mountain marathon. According to the most up-to-date pre-analytical warnings, venous blood was collected in K2EDTA tubes (BD Vacutainer^®^, Becton Dickinson, Franklin Lakes, NJ, USA), and plasma, obtained following centrifugation at standard conditions of 1300× *g* for 10 min at room temperature (22 °C), was immediately frozen at −80 °C until assayed.

### 2.3. miRNA Profiling

miRNA profiling analysis was performed accordingly [20]. Briefly, miRNA-enriched total RNA fraction was isolated from equal aliquot of plasma pooled from each subject using miRCURY™ RNA Isolation Kit (Exiqon A/S, Vedbaek, Denmark); isolation efficiency was checked with synthetic oligonucleotides added at known concentrations (spike-in: UniSp2, UniSp4, UniSp5). Reverse transcription reaction was conducted with miRCURY LNA™ Universal RT miRNA PCR. UniSp6 and cel-39 were used as reaction controls. qPCR was performed on a StepOne Plus (Applied Biosystem, Foster City, CA, USA) using serum/plasma miRCURY LNA™ miRNA focus panel and ExiLENT SYBR Green Master Mix (Exiqon). The qPCR reaction was conducted as follows: polymerase activation for 10 min at 95 °C, amplification (40 cycles: 10 s at 95 °C and 60 s at 60 °C) and melting curves. The analytical output, expressed as quantification cycle (Cq), was analyzed with GenEx software v6 (Exiqon). Three inter-plate (IPC) calibrators, with specific primer pairs and DNA template, were used to adjust Cq values from different plate runs of different experiments. Only miRNAs with a Cq <37 were further considered in the analysis. The 2^−ΔΔCT^ method was applied to calculate the relative expression of each miRNA, using hsa-miR-320d as normalizers. This miRNA was identified as the most appropriate based on a study of miRNA stability, as previously reported [20]. Hemolysis was checked by hsa-miR-23-a-to-hsa-miR-451 ΔCq difference (positive if >7). Analyses were conducted in triplicate.

### 2.4. Statistical Analysis

Given their distribution, data from the two cohorts were compared throughout a parametric unpaired t-test with Welch’s correction. Differences were considered significant when *p* < 0.05 (Prism^®^ v6.01, GraphPad Software, La Jolla, CA, USA). Only miRNAs with a significant (*p* < 0.05) fold change ≥ ±2 were considered.

### 2.5. Article Selection

Published articles on circulating miRNAs associated with cancer were selected on PubMed by searching for “serum miRNAs”, “serum microRNAs”, “plasma miRNAs”, “plasma microRNAs”, “circulating miRNAs”, “circulating microRNAs” with “cancer diagnosis” for five cancers: lung cancer (LC), prostate cancer (PC), colon cancer (CC), liver cancer (HC) and gastroesophageal cancer (GEC) and related subtypes. These cancers were chosen as the five most frequent cancers in males worldwide, according to the “Global Cancer Observatory” (https://gco.iarc.fr/, accessed on 31 December 2020), for which exist evidence, through meta-analysis (published from 2010 to 2020), on association with physical activity (all-cancer risk/mortality [25,26,27,28,29]; LC [30,31]; PC [32]; CC [33,34,35]; HC [36]; GEC [37,38,39,40,41,42,43,44,45]). Only studies on human, published from 2010, and demonstrating a clinical relevance for single serum/plasma miRNA or serum/plasma miRNA signatures including at least one of the modulated miRNAs in our trained cohort were considered. The clinical relevance was defined only in the presence of a literature-derived ROC curve analysis resulting in an area under curve (AUC) >0.7, for diagnostic purpose [46]. This search referred to all 179 analyzed miRNAs.

## 3. Results

### 3.1. Selection of Cancer-Associated miRNAs

**Figure 1:**
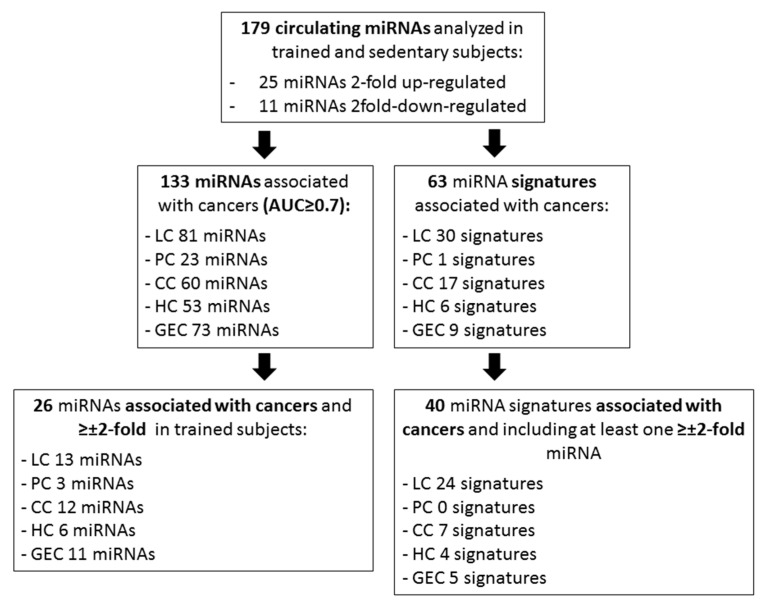
Description of selection strategy of cancer associated miRNAs and signatures.

The selection strategy of cancer-associated miRNAs and signatures is summarized in Figure 1.

The expression profile of a panel of 179 miRNAs (Table S1), the most abundant in circulation, were analyzed in plasma of two cohorts: sedentary and trained cohorts. Thirty-six miRNAs were significantly modulated by PA: 25 miRNAs were ≥2-fold upregulated and 11 were ≥2-fold downregulated (Table 1).

As shown in Table 2, among the 179 analyzed miRNAs, 133 have been regarded as of diagnostic usefulness for at least one of the five most prevalent cancers: 81 for LC (Table S2), 23 for PC (Table S3), 60 for CC (Table S4), 53 for HC (Table S5) and 73 for GEC (Table S6). Twenty-six miRNAs were significantly ≥2-fold upregulated (15 miRNAs) or ≥2-fold downregulated (11 miRNAs) in trained subjects, compared to sedentary subjects (Table 2).

Several miRNA signatures, associated with the considered cancers, made up by miRNAs included in our panel, have been also considered (Table 3): 30 for LC (Table S2), 1 for PC (Table S3), 17 for CC (Table S4), 6 for HC (Table S5) and 9 for GEC (Table S6). Forty signatures included at least one miRNA that resulted modulated by PA in our experimental setting (Table 3).

### 3.2. Effects of the Physical Activity Status on the Circulating Levels of miRNAs Associated with Cancers

#### 3.2.1. Lung Cancer

Among the 179 miRNAs, a diagnostic potential in LC and subtypes has been established for 81 miRNAs (Table S2). Of these, 13 resulted modulated by PA in our experimental setting: eight miRNAs were ≥2-fold upregulated (hsa-miR-155-5p, hsa-miR-16-2-3p, hsa-miR-20b-5p, hsa-miR-30e-3p, hsa-miR-320a, hsa-miR-320b, hsa-miR-409-3p and hsa-miR-93-3p) and five were ≥2-fold downregulated (hsa-let-7a-5p, hsa-miR-126-3p, hsa-miR-140-5p, hsa-miR-181a-5p and has-miR-199a-5p) (Table 4).

has-miR-155-5p, hsa-miR-16-2-3p, hsa-miR-20b-5p, hsa-miR-30e-3p, hsa-miR-320a, hsa-miR-320b, hsa-miR-93-3p, hsa-let-7a-5p and hsa-miR-126-3p have been described as discriminating non-small cell lung cancer (NSCLC) patients from healthy individuals. Other miRNAs associated with LC diagnosis (hsa-miR-155-5p in LC; hsa-miR-126-3p, hsa-miR-140-5p and hsa-miR-409-3p in lung adenocarcinoma (LA); and hsa-miR-181a-5p in lung squamous cell carcinoma (LSCC)) were also affected by the training status. Additionally, miRNA included in the analyzed panel were found in 30 miRNA signatures associated with LC diagnosis (Table 3). Twenty-four signatures diagnostic for LC or LC subtypes, included at least one of the 13 miRNAs modulated in the trained cohort: twenty signatures included one of the thirteen miRNAs that resulted modulated in trained subjects, two signatures included two modulated miRNAs and two signatures included three modulated miRNAs.

#### 3.2.2. Prostate Cancer

Twenty-three miRNAs, within the analyzed panel, have a diagnostic potential in PC (Table S2). Of these, two miRNAs (hsa-miR-326 and hsa-miR-874a-3p) were found ≥2-fold significantly upregulated and one (hsa-miR-93-3p) downregulated in trained subjects (Table 4). No miRNA signatures associated with PC diagnosis, including miRNAs modulated in trained individuals, were identified (Table 3).

#### 3.2.3. Colon Cancer

Sixty miRNAs, within the analyzed panel, have a diagnostic potential in CC and CRC (Table S4). Of these, 12 were modulated in our experimental setting by PA: hsa-miR-127-3p, hsa-miR-155-5p, hsa-miR-320a, hsa-miR-335a-3p and hsa-miR-93-3p were ≥2-fold significantly upregulated, while hsa-let-7a-5p, hsa-miR-181a-5p, hsa-miR-18b-5p, hsa-miR-199a-5p, hsa-miR-29a-3p, hsa-miR-378a-3p and hsa-miR-424-5p were ≥2-fold significantly downregulated in trained subjects (Table 4). Seventeen miRNA signatures, with diagnostic potential for CC or CRC (Table 3), including several of the 179 analyzed circulating miRNAs, were identified in the literature. Among them, four signatures included one of the eleven miRNAs affected by the trained status and three signatures included two modulated miRNAs (Table 3).

#### 3.2.4. Liver Cancer

Fifty-three miRNAs, within the analyzed panel, have a diagnostic potential in HC (Table S5). Of these, six miRNAs were modulated in our experimental setting by physical activity: hsa-miR-29b-3p and hsa-miR-423-3p were ≥2-fold significantly upregulated, while hsa-miR-126-3p, hsa-miR-181a-5p, hsa-miR-29a-3p and hsa-miR-574-3p were ≥2-fold significantly downregulated in trained individuals (Table 4). Six miRNA signatures associated with HC diagnosis include analyzed miRNAs (Table 3): four signatures include one miRNA modulated in trained subjects compared to sedentary subjects.

#### 3.2.5. Gastroesophageal Cancer

Seventy-three miRNAs, within the analyzed panel, have a diagnostic potential for GEC (Table S6). Of these, 11 miRNAs were modulated in trained subject compared to sedentary subjects: hsa-miR-127-3p, hsa-miR-16-2-3p, hsa-miR-20b-5p, hsa-miR-629-5p and hsa-miR-93-3p were ≥2-fold significantly upregulated, while hsa-let-7a-5p, hsa-let-7d-3p, hsa-miR-140-5p, hsa-miR-199a-5p, hsa-miR-27b-3p and hsa-miR-378a-3p were ≥2-fold significantly downregulated in trained individuals (Table 4). More specifically, hsa-let-7a-5p, hsa-let-7d-3p, hsa-20b-5p and hsa-miR-93-3p have been described as discriminating between esophageal squamous cell carcinoma (ESCC) patients and healthy individuals, while hsa-miR-140-5p, hsa-miR-16-2-3p, hsa-miR-199a-5p, hsa-miR-27b-3p, hsa-miR-378a-3p, hsa-miR-629-5p and hsa-miR-93-3p are diagnostic for gastric cancer (GC). Among the nine miRNA signatures associated with GEC diagnosis, five signatures include one miRNA that resulted modulated in trained individuals (Table 3).

## 4. Discussion

Several circulating miRNAs, both singularly and grouped in signatures, have been associated with cancer development risk, detection and progression [13]. In parallel, many studies have demonstrated that regular PA reduces the global risk of developing a cancer as well as improves prognosis and reduces the risk of metastasis and side effects of the therapy in subjects treated for primitive tumor [17,94,95]. However, the impact of PA on circulating miRNAs associated with cancer is mostly neglected, as recently reviewed in [21]. Therefore, this study provides first evidence about the alteration in the circulating level of cancer associated miRNAs in response to chronic PA.

The main question this study aimed to address is whether physical active status may represent a modifier of the baseline circulating miRNA profile and, hence, may act as a pre-analytical variable that affect the diagnostic potential of miRNAs associated with cancer. In our study, a panel of 179 circulating miRNAs was analyzed. First, for each miRNA, the association with the five most frequent cancers in males (LC, PC, CC, HC and GEC) was investigated; only those miRNAs with an established diagnostic potential, defined as the ability to discriminate between cancer patients and healthy subjects and associated to a significant AUC ≥ 0.7, in ROC analysis, was considered. Based on this assumption, we identified 133 miRNAs. Second, the expression profile of the 179-miRNA panel was determined in plasma samples of highly trained subjects and their sedentary counterparts. Only miRNA significantly ≥2 fold up- or downregulated in trained subjects, compared to sedentary subjects, were further investigated. As expected, our two cohorts are featured by a different miRNA profile: the expression level of 36 miRNAs (25 upregulated and 11 downregulated) was significantly (*p* < 0.05) and relevantly (≥2 fold) altered in physically active subjects (Table 1). Among these, 26 miRNAs (15 upregulated and 11 downregulated) have been previously associated with at least one of the most frequent cancers in males (see the tables in the Supplementary Materials). Of the 15 upregulated miRNAs, eight have been associated with LC, two with PC and HC and five with CC and GEC. Of the 11 downregulated miRNAs, five have been associated with LC, one with PC, seven with CC, four with HC and six with GEC. These results demonstrate that the adaptation to regular exercise determines a baseline modification of several circulating miRNAs and this fact, if not considered, may limit their clinical applicability, thus confirming previous hypothesis [12]. Similarly, this consideration can be extended to miRNA signatures since 19 miRNAs that resulted significantly modulated by PA are included in diagnostic miRNA signatures from the literature. miRNA signature means a series of miRNAs that have an overall diagnostic potential. A diagnostic miRNA signature does not necessarily include all single miRNA found with diagnostic potential in a specific study; a miRNA signature can also include miRNAs that, singularly, do not have a diagnostic potential. Moreover, a signature can include both miRNAs up- and -downregulated in a specific experimental setting. In this study, signatures from the literature that include at least one of the modulated miRNAs in our trained cohort were considered. Based on our literature search, 63 signatures were identified. Of the signatures that include at least one modulated miRNA, in our experimental setting, 24 are associated with LC diagnosis, 0 with PC, 7 with CC, 4 with HC and 5 with GEC. Thereby, the presence of miRNAs affected by physical active status within a diagnostic signature may reduce its clinical reliability. Consequently, the greater is the number of miRNAs modulated by PA within a signature, the lower is the clinical reliability of the signature itself.

From these results, the training status-dependent alterations of the circulating levels of cancer-associated miRNAs in healthy individuals are remarkable. Importantly, 107 miRNAs (of the 133 circulating miRNAs associated with the five cancers) and 23 signatures resulted not affected by regular training, and, given their robustness, the analysis of these miRNAs or signatures might be privileged. Alterations of hematological and biochemical parameters in response to chronic PA are well documented [22], as well as, although to a lesser extent, for miRNAs [12]. Chronic PA alters the level of miRNAs involved in skeletal muscle and bone metabolisms [20], inflammation, immunity and angiogenesis [96], as well as those associated with metabolic syndrome and diabetes [97], cardiovascular disease [98], skeletal muscle and bone diseases [9,99]. However, the measurement of these indices is challenging. Sample-associated and subject-associated pre-analytical variables must be considered. The former includes patient preparation (timing and posture), sample collection (sample identification and labeling, type of disposables, order of draw, phlebotomy procedure and contamination), transportation (length and environmental conditions), preparation (centrifugation length, speed and temperature and aliquot preparation) and storage (length, temperature and freeze–thaw cycles) [100]. Subject-associated variables, besides the uncontrollable but considerable biological ones (e.g., age, gender, ethnicity, diseases and circannual/seasonal rhythm), include medications, physical activity status (acute and chronic) and menstrual cycle phase, based on which the sampling can be opportunely temporized [101]. As we recently demonstrated for the same set of miRNAs, many of these variables can affect their determination [102,103], and, according to the present study, the physical activity status must also be considered. Therefore, a strict definition of the pre-analytical variables affecting circulating miRNAs measurement, including PA, is needed to allow a correct interpretation of the final analytical output.

Although based on a solid experimental approach relying on a meticulous control of the pre-analytical phase, and a robust post-analytical data analysis based on a well-defined normalization strategy, this study suffers some limitations that should be taken into consideration. First, the identification of miRNAs and miRNA signatures associated with cancer diagnosis is based on a literature search that, although extensive and systematically conducted, may be not fully exhaustive. In this regard, since the rather not exhaustive information available, it was not possible to select the research articles taking into consideration important variables such as gender, age, medications and comorbidities. Second, different studies may have differently scored a miRNA, and, hence, although the criterion of AUC > 0.7 has been established, the actual diagnostic relevance of certain miRNAs may have been under- or overestimated. Third, due to the discrepancies among the studies the limit of having considered healthy subjects, it was not possible to determine whether PA may play a protective role against cancers, other than its only pre-analytical effect. Finally, the sample size could be considered rather small for a case–control study. However, this limitation would be, at least partially, overcome thanks to the very high homogeneity level of the subjects within each of the two cohorts in terms of subject-specific pre-analytical variables (e.g., age range and avoidance from physical activity for the sedentary cohort and physical activity level and training status for trained subjects).

## 5. Conclusions

Due to the PA contribution to alteration of circulating level of miRNAs, many of which are associated with cancer diagnostic, this study emphasized PA as a pre-analytical variable. The consistent variability of circulating miRNA induced by PA is of great relevance in diagnostics since miRNA variability may reflect the adaptive response of the whole organism to the PA status, rather than the presence of a pathological condition, as cancer. Therefore, further studies are needed to better standardize the PA effects in order to improve the clinical implementation of circulating miRNAs.

## Figures and Tables

**Table 1:** Fold-Change analyzed miRNAs significantly ≥2-fold up- or downregulated in trained subjects compared to sedentary subject (*p*-value < 0.05).

miRNAs	Fold-Change	*p*-Value
hsa-let-7a-5p	0.147	0.035
hsa-let-7d-3p	0.327	0.012
hsa-miR-1	32.146	0.003
hsa-miR-126-3p	0.494	<0.001
hsa-miR-127-3p	33.726	0.034
hsa-miR-140-5p	0.016	0.002
hsa-miR-148b-3p	32.664	0.013
hsa-miR-155-5p	32.980	<0.001
hsa-miR-16-2-3p	34.059	0.028
hsa-miR-181a-5p	0.015	<0.001
hsa-miR-186-5p	33.453	0.028
hsa-miR-199a-5p	0.008	0.022
hsa-miR-20b-5p	33.930	0.037
hsa-miR-27b-3p	0.409	0.015
hsa-miR-28-3p	33.374	0.001
hsa-miR-29a-3p	0.015	<0.001
hsa-miR-29b-3p	34.476	0.005
hsa-miR-30e-3p	32.977	<0.001
hsa-miR-320a	2.378	0.018
hsa-miR-320b	3.037	0.013
hsa-miR-326	34.710	0.0027
hsa-miR-335-3p	33.395	0.027
hsa-miR-376a-3p	32.728	0.015
hsa-miR-378a-3p	0.031	0.014
hsa-miR-382-5p	33.174	0.001
hsa-miR-409-3p	35.541	<0.001
hsa-miR-421	32.351	0.007
hsa-miR-423-3p	33.454	0.028
hsa-miR-424-5p	0.031	0.015
hsa-miR-495-3p	32.963	0.019
hsa-miR-502-3p	32.979	0.019
hsa-miR-505-3p	33.851	0.036
hsa-miR-574-3p	0.256	0.002
hsa-miR-629-5p	34.614	0.001
hsa-miR-874-3p	32.953	0.019
hsa-miR-93-3p	32.869	0.017

**Table 2:** List of all miRNAs, among the 179 assayed miRNAs, with diagnostic potential for at least one of the five most prevalent cancers (lung cancer, prostate cancer, colon cancer, liver cancer and gastro-esophageal cancer). In bold are reported miRNAs significantly (*p*-value < 0.05) ≥2-fold up- or downregulated in trained subjects compared to sedentary subject.

miRNAs	Fold-Change	*p*-Value
**hsa-let-7a-5p**	**0.147**	**0.035**
hsa-let-7b-3p	Undetected	
hsa-let-7b-5p	1.508	0.177
hsa-let-7c-5p	0.626	0.417
**hsa-let-7d-3p**	**0.327**	**0.012**
hsa-let-7d-5p	1.663	0.393
hsa-let-7e-5p	0.530	0.008
hsa-let-7f-5p	0.348	0.057
hsa-let-7g-5p	0.571	0.372
hsa-let-7i-5p	1.363	0.705
hsa-miR-100-5p	Undetected	
hsa-miR-101-3p	0.197	0.128
miR-103a-3p	0.113	0.275
miR-106a-5p	0.639	0.461
hsa-miR-106b-3p	Undetected	
hsa-miR-106b-5p	0.638	0.586
hsa-miR-107	0.121	0.219
hsa-miR-10b-5p	Undetected	
hsa-miR-122-5p	0.104	0.139
miR-125a-5p	0.508	0.172
hsa-miR-125b-5p	0.010	0.215
**hsa-miR-126-3p**	**0.494**	**<0.001**
hsa-miR-126-5p	1.287	0.692
**hsa-miR-127-3p**	**33.726**	**0.034**
hsa-miR-130a-3p	44.819	0.054
hsa-miR-130b-3p	46.322	0.068
hsa-miR-133a-3p	Undetected	
hsa-miR-139-5p	0.373	0.139
**hsa-miR-140-5p**	**0.016**	**0.002**
hsa-miR-141-3p	Undetected	
hsa-miR-142-3p	0.081	0.133
hsa-miR-142-5p	0.456	0.120
hsa-miR-143-3p	Undetected	
hsa-miR-144-3p	0.077	0.352
hsa-miR-144-5p	47.871	0.088
hsa-miR-145-5p	0.501	0.104
hsa-miR-146a-5p	0.859	0.744
hsa-miR-146b-5p	0.821	0.449
hsa-miR-148a-3p	0.336	0.304
**hsa-miR-148b-3p**	**32.664**	**0.013**
miR-150-5p	0.998	0.995
hsa-miR-151a-3p	2.649	0.423
hsa-miR-151a-5p	0.355	0.233
hsa-miR-152-3p	0.670	0.515
hsa-miR-154-5p	Undetected	
**hsa-miR-155-5p**	**32.980**	**<0.001**
hsa-miR-15a-5p	1.045	0.965
hsa-miR-15b-3p	0.756	0.380
hsa-miR-15b-5p	2.689	0.493
**hsa-miR-16-2-3p**	**34.059**	**0.028**
hsa-miR-16-5p	1.246	0.537
hsa-miR-17-5p	4.460	0.544
**hsa-miR-181a-5p**	**0.015**	**<0.001**
hsa-miR-18a-5p	0.813	0.666
hsa-miR-18b-5p	0.315	0.284
hsa-miR-191-5p	1.512	0.577
hsa-miR-192-5p	0.623	0.262
hsa-miR-193a-5p	Undetected	
hsa-miR-194-5p	0.934	0.771
hsa-miR-195-5p	Undetected	
hsa-miR-197-3p	1.382	0.377
hsa-miR-199a-3p	0.831	0.425
**hsa-miR-199a-5p**	**0.008**	**0.022**
hsa-miR-19a-3p	0.355	0.226
hsa-miR-19b-3p	0.287	0.195
hsa-miR-20a-5p	0.672	0.526
**hsa-miR-20b-5p**	**33.930**	**0.037**
hsa-miR-200a-3p	Undetected	
hsa-miR-200c-3p	53.405	0.247
hsa-miR-205-5p	Undetected	
hsa-miR-21-5p	0.969	0.896
hsa-miR-210-3p	Undetected	
hsa-miR-215-5p	0.371	0.127
hsa-miR-22-3p	0.383	0.156
hsa-miR-22-5p	Undetected	
hsa-miR-221-3p	0.220	0.140
hsa-miR-222-3p	0.699	0.409
hsa-miR-223-3p	1.005	0.990
hsa-miR-223-5p	0.567	0.290
hsa-miR-23a-3p	0.994	0.564
hsa-miR-23b-3p	1.674	0.428
hsa-miR-24-3p	1.329	0.419
hsa-miR-25-3p	1.311	0.443
hsa-miR-26a-5p	1.436	0.717
hsa-miR-27a-3p	3.069	0.276
**hsa-miR-27b-3p**	**0.409**	**0.015**
**hsa-miR-29a-3p**	**0.015**	**<0.001**
**hsa-miR-29b-3p**	**34.476**	**<0.001**
hsa-miR-29c-3p	66.131	0.185
hsa-miR-30a-5p	Undetected	
hsa-miR-30b-5p	0.291	0.194
hsa-miR-30c-5p	0.188	0.083
hsa-miR-30d-5p	1.656	0.180
**hsa-miR-30e-3p**	**32.977**	**<0.001**
hsa-miR-30e-5p	0.377	0.126
hsa-miR-301a-3p	0.184	0.090
**hsa-miR-320a**	**2.378**	**0.018**
**hsa-miR-320b**	**3.037**	**0.013**
hsa-miR-320c	3.268	0.203
hsa-miR-324-3p	0.561	0.086
**hsa-miR-326**	**34.710**	**0.003**
hsa-miR-328-3p	1.154	0.868
hsa-miR-331-3p	0.010	0.203
**hsa-miR-335-3p**	**33.395**	**0.027**
hsa-miR-335-5p	Undetected	
hsa-miR-339-3p	Undetected	
hsa-miR-339-5p	0.534	0.194
hsa-miR-342-3p	0.541	0.308
hsa-miR-34a-5p	Undetected	
hsa-miR-375	0.326	0.286
hsa-miR-376c-3p	44.991	0.183
**hsa-miR-378a-3p**	**0.031**	**0.014**
**hsa-miR-409-3p**	**35.541**	**<0.001**
**hsa-miR-423-3p**	**33.454**	**0.028**
hsa-miR-423-5p	1.582	0.281
**hsa-miR-424-5p**	**0.031**	**0.015**
hsa-miR-425-3p	45.356	0.077
hsa-miR-425-5p	1.364	0.590
hsa-miR-451a	0.213	0.351
hsa-miR-483-5p	0.678	0.466
hsa-miR-484	0.491	0.168
hsa-miR-486-5p	4.640	0.173
hsa-miR-497-5p	Undetected	
**hsa-miR-574-3p**	**0.256**	**0.002**
**hsa-miR-629-5p**	**34.614**	**0.001**
hsa-miR-7-1-3p	Undetected	
hsa-miR-7-5p	Undetected	
**hsa-miR-874-3p**	**46.335**	**0.019**
hsa-miR-885-5p	Undetected	
hsa-miR-92a-3p	4.977	0.221
hsa-miR-92b-3p	Undetected	
**hsa-miR-93-3p**	**32.869**	**0.017**
hsa-miR-93-5p	0.570	0.377

**Table 3:** List of diagnostic signatures for lung (LC), prostate (PC), colon (CC), liver (HC) and gastro-esophageal (GEC) cancers that include miRNAs significantly ≥2-fold up- or downregulated in trained subjects compared to sedentary subject (*p*-value < 0.05).

Cancer	Signature	miRNA (FC ≥ ±2, *p* < 0.05)	References
LC	miR-194-5p, miR-451a, miR-486-5p		[47]
	miR-21, miR-339-5p		[48]
	let-7b-5p, let-7e-5p, miR-21-5p, miR-24-3p		[49]
	miR-15b-5p, miR-16-5p, miR-20a-5p		[50]
	miR-146b, miR-205, miR-29c, miR-30b		[51]
	miR-145, miR-21		[52]
	miR-19b-3p, miR-21-5p, miR-221-3p, miR-409-3p, miR-425-5p, miR-584-5p	miR-409-3p	[53]
	miR-142-5p, miR-146a-5p, miR-223-3p, miR-409-3p	miR-409-3p	[54]
	miR-106a-5p, miR-181a-5p, miR-21-5p, miR-93-5p	miR-181a-5p	[55]
	miR-126-3p, miR-145, miR-205-5p, miR-210-3p	miR-126-3p	[56]
	miR-16, miR-205, miR-486	miR-486	[57]
	miR-126, miR-21, miR-210, miR-486-5p	miR-126	[58]
	let-7a, miR-21	let-7a	[59]
	miR-125a-5p, miR-126, miR-25	miR-126	[60]
	miR-148a, miR-148b, miR-152, miR-21	miR-148b	[61]
	miR-155, miR-21	miR-155	[52]
	miR-145, miR-155	miR-155	[52]
	miR-140-5p, miR-92a	miR-140-5p	[62]
	miR-140-5p, miR-331-3p, miR-92a	miR-140-5p	[62]
	miR-92a, miR-140-5p, miR-331-3p, miR-374a	miR-140-5p	[62]
	miR-140-5p, miR-223, miR-331-3p, miR-374a, miR-92a	miR-140-5p	[62]
	miR-140-5p, miR-148a, miR-223 miR-331-3p, miR-374a, miR-92a	miR-140-5p	[62]
	miR-140-5p, miR-148a, miR-223 miR-331-3p, miR-374a, miR-484, miR-92a	miR-140-5p	[62]
	miR-140-5p, miR-148a, miR-223, miR-328, miR-331-3p, miR-374a, miR-484, miR-92a	miR-140-5p	[62]
	miR-140-5p, miR-148a, miR-191, miR-223, miR-328, miR-331-3p, miR-374a, miR-484, miR-92a	miR-140-5p	[62]
	miR-140-5p, miR-148a, miR-191, miR-223, miR-30c, miR-328, miR-331-3p, miR-374a, miR-484, miR-92a	miR-140-5p	[62]
	miR-145, miR-152, miR-199a, miR-20a, miR-221, miR-222, miR-223, miR-24, miR-25, miR-320	miR-199a, miR-320	[63]
	miR-140-5p, miR-148a, miR-191, miR-223, miR-29a, miR-30c, miR-328, miR-331-3p, miR-374a, miR-484, miR-92a	miR-140-5p, miR-29a	[62]
	let-7d, miR-140-5p, miR-148a, miR-191, miR-223, miR-29a, miR-30c, miR-328, miR-331-3p, miR-374a, miR-484, miR-92a	let-7d, miR-140-5p, miR-29a	[62]
	let-7d, miR-140-5p, miR-148a, miR-191, miR-223, miR-29a, miR-30c, miR-328, miR-331-3p, miR-374a, miR-484, miR-92a	let-7d, miR-140-5p, miR-29a	[62]
PC	miR-21, and miR-221		[64]
CC	miR-23a-3p, miR-27a-3p, miR-142-5p, miR-376c-3p		[65]
	miR-19a-3p, miR-21-5p, miR-425-5p		[66]
	miR-21, miR-221, miR-92a		[67]
	miR-21, miR-92a		[68]
	miR-130a, miR-27a		[69]
	miR-18a, miR-21, miR-22, miR-25		[70]
	miR-18a, miR-191, miR-221, miR-223, miR-24, miR-92a		[71]
	miR-223, miR-24, miR-92a		[71]
	miR-19a, miR-19b		[72]
	miR-19a, miR-19b, miR-15b		[72]
	miR-17, miR-21, miR-29a, miR-92	miR-29a	[73]
	miR-125b, miR-21, miR-29a	miR-29a	[74]
	miR-29a, miR-92a	miR-29a	[75]
	miR-24, miR-320a, miR-423-5p	miR-320a	[76]
	miR-15b, miR-18a, miR-19a, miR-19b, miR-29a, miR-335	miR-29a, miR-335	[77]
	miR-155, miR-21, miR-200c, miR-210, miR-29a	miR-155, miR-29a	[67]
	miR-409-3p, miR-7, miR-93	miR-409-3p, miR-93	[78]
HC	miR-101, miR-21, miR-26a		[79]
	miR-125b, miR-223, miR-26a, miR-27a		[80]
	miR-10b, miR-106b, miR-181a	miR-181a	[81]
	mir-192-5p, miR-27b-3p	miR-27b-3p	[82]
	mir-122, miR-29b, mir-885-5p	miR-29b	[83]
	miR-23a, miR-23b, miR-342-3p, miR-423	miR-423	[84]
GEC	miR-106a-5p, miR-17-5p, miR-19b-3p, miR-30a-5p		[85]
	miR-106a, miR-17		[86]
	miR-223, miR-375		[87]
	miR-1, miR-20a, miR-27a, miR-34, miR-423-5p		[88]
	miR-106a, miR-106b, miR-21, miR-93	miR-93	[89]
	miR-629, miR-652	miR-629	[90]
	miR-16, miR-25, miR-451, miR-486-5p, miR-92a	miR-16	[91]
	miR-107, miR-144, miR-152, miR-21, miR-342, miR-93	miR-93	[92]
	miR-133a-3p, miR-382-5p, miR-451a	miR-382-5p	[93]

**Table 4:** List of miRNAs significantly ≥2-fold up- or downregulated in trained subjects compared to sedentary subject (*p*-value < 0.05) and with diagnostic potential for lung (LC), prostate (PC), colon (CC), liver (HC) and gastro-esophageal (GEC) cancers. The modulation of each miRNA, in trained subjects compared to sedentary subjects, is reported as ↑ (upregulated), ↓ (downregulated).

Cancer	miRNAs	FC ≥ ±2 & *p* < 0.05
LC	hsa-let-7a-5p	↓
	hsa-miR-126-3p	↓
	hsa-miR-140-5p	↓
	hsa-miR-155-5p	↑
	hsa-miR-16-2-3p	↑
	hsa-miR-181a-5p	↓
	hsa-miR-199a-5p	↓
	hsa-miR-20b-5p	↑
	hsa-miR-30e-3p	↑
	hsa-miR-320a	↑
	hsa-miR-320b	↑
	hsa-miR-409-3p	↑
	hsa-miR-93-3p	↑
PC	hsa-miR-326	↑
	hsa-miR-874-3p	↑
	hsa-miR-93-3p	↓
CC	hsa-let-7a-5p	↓
	hsa-miR-127-3p	↑
	hsa-miR-155-5p	↑
	hsa-miR-181a-5p	↓
	hsa-miR-18b-5p	↓
	hsa-miR-199a-5p	↓
	hsa-miR-29a-3p	↓
	hsa-miR-320a	↑
	hsa-miR-335a-3p	↑
	hsa-miR-378a-3p	↓
	hsa-miR-424-5p	↓
	hsa-miR-93-3p	↑
HC	hsa-miR-126-3p	↓
	hsa-miR-181a-5p	↓
	hsa-miR-29a-3p	↓
	hsa-miR-29b-3p	↑
	hsa-miR-423-3p	↑
	hsa-miR-574-3p	↓
GEC	hsa-let-7a-5p	↓
	hsa-let-7d-3p	↓
	hsa-miR-127-3p	↑
	hsa-miR-140-5p	↓
	hsa-miR-16-2-3p	↑
	hsa-miR-199a-5p	↓
	hsa-miR-20b-5p	↑
	hsa-miR-27b-3p	↓
	hsa-miR-378a-3p	↓
	hsa-miR-629-5p	↑
	hsa-miR-93-3p	↑

## Data Availability

Raw data can be found at https://zenodo.org/record/4058363#.YC4d5I9KiM8, accessed on 30 November 2020.

## Supplementary Materials

The following are available online at https://www.mdpi.com/article/10.3390/diagnostics11050820/s1. Table S1: Fold-change of the 179 miRNAs analyzed in trained subjects compare with sedentary subjects. In bold are reported miRNAs significantly ≥2-fold up- or downregulated in trained subjects compared to sedentary subject (*p*-value > 0.05). Table S2: List of the miRNAs and miRNA signature associated with diagnosis in lung cancer from the literature. Only miRNAs with significant clinically relevance (AUC ≥ 0.7) and signatures with at least one modulated miRNA from our experimental setting were considered. Table S3: List of the miRNAs and miRNA signature associated with diagnosis in prostate cancer from the literature. Only miRNAs with significant clinically relevance (AUC ≥ 0.7) and signatures with at least one modulated miRNA from our experimental setting were considered. Table S4: List of the miRNAs and miRNA signatures associated with diagnosis in colon cancer from the literature. Only miRNAs with significant clinically relevance (AUC ≥ 0.7) and signature with at least one modulated miRNA from our experimental setting were considered. Table S5: List of the miRNAs and miRNA signatures associated with diagnosis in liver cancer from the literature. Only miRNAs with significant clinically relevance (AUC ≥ 0.7) and signature with at least one modulated miRNA from our experimental setting were considered. Table S6: List of the miRNAs and miRNA signature associated with diagnosis in gastro-esophageal cancer from the literature. Only miRNAs with significant clinically relevance (AUC ≥ 0.7) and signatures with at least one modulated miRNA from our experimental setting were considered.
