# Supplementary material for: A Physically Active Status Affects the Circulating Profile of Cancer-Associated miRNAs
Source: Diagnostics (Basel). 2021 Apr 30;11(5):820. doi: 10.3390/diagnostics11050820 (PMC8147229; doi:10.3390/diagnostics11050820)
Supplement: Supplementary file 1 [file diagnostics-11-00820-s001.zip › Supplementary Table S1.pdf]

diagnostics

Article

A Physically Active Status Affects the Circulating Profile of Cancer-Associated miRNAs

Martina Faraldi [1], Laura Gerosa [2,*], Marta Gomarasca [1], Veronica Sansoni [1], Silvia Perego [1], Ewa Ziemann [3], Giuseppe Banfi [1,4] and Giovanni Lombardi [1,3]

[1] IRCCS Istituto Ortopedico Galeazzi, Laboratory of Experimental Biochemistry & Molecular Biology, 20161 Milano, Italy; martina.faraldi@grupposandonato.it (M.F.); marta.gomarasca@grupposandonato.it (M.G.); veronica.sansoni@grupposandonato.it (V.S.); silvia.perego@grupposandonato.it (S.P.); banfi.giuseppe@unisr.it (G.B.); giovanni.lombardi@grupposandonato.it (G.L.)
[2] Gruppo San Donato Foundation, 20122 Milano, Italy
[3] Department of Athletics, Strength and Conditioning, Poznań University of Physical Education, 61-871 Poznań, Poland; ziemann@awf.poznan.pl
[4] Vita-Salute San Raffaele University, 20132 Milano, Italy
* Correspondence: laura.gerosa@grupposandonato.it; Tel.: +39-02166214068

Citation: Faraldi, M.; Gerosa, L.; Gomarasca, M.; Sansoni, V.; Perego, S.; Ziemann, E.; Banfi, G.; Lombardi, G. A Physically Active Status Affects the Circulating Profile of Cancer-Associated miRNAs. *Diagnostics* **2021**, *11*, 820. https://doi.org/10.3390/diagnostics11050820

Academic Editor: Anastasia Malek

Received: 24 March 2021
Accepted: 29 April 2021
Published: 30 April 2021

Publisher's Note: MDPI stays neutral with regard to jurisdictional claims in published maps and institutional affiliations.

Copyright: © 2021 by the authors. Licensee MDPI, Basel, Switzerland. This article is an open access article distributed under the terms and conditions of the Creative Commons Attribution (CC BY) license (http://creativecommons.org/licenses/by/4.0/).

Supplementary Table S1. Fold-Change of the 179 miRNAs analyzed in trained subjects compared with sedentary subjects. In bold are reported miRNAs significantly ≥±2-fold up- or down-regulated in trained subjects compared with sedentary subject (p-value <0.05).

miRNAs	Fold-Change	*p*-value
**hsa-let-7a-5p**	**0. 147**	**0.0346**
hsa-let-7b-3p	Undetected	
hsa-let-7b-5p	1.508	0.1766
hsa-let-7c-5p	0.626	0.4174
**hsa-let-7d-3p**	**0.327**	**0.0124**
hsa-let-7d-5p	1.663	0.3932
hsa-let-7e-5p	0.530	0.0076
hsa-let-7f-5p	0.348	0.0566
hsa-let-7g-5p	0.571	0.3725
hsa-let-7i-5p	1.363	0.7046
**hsa-miR-1**	**32.146**	**0.0030**
hsa-miR-100-5p	Undetected	
hsa-miR-101-3p	0.197	0.1283
hsa-miR-103a-3p	0.113	0.2749
hsa-miR-106a-5p	0.639	0.4608
hsa-miR-106b-3p	Undetected	
hsa-miR-106b-5p	0.638	0.5860
hsa-miR-107	0.121	0.2193
hsa-miR-10b-5p	Undetected	
hsa-miR-122-5p	0.104	0.1387
hsa-miR-125a-5p	0.508	0.1720
hsa-miR-125b-5p	0.010	0.2153
hsa-miR-1260a	65.646	0.1806
**hsa-miR-126-3p**	**0.494**	**0.0001**
hsa-miR-126-5p	1.287	0.6920
**hsa-miR-127-3p**	**33.726**	**0.0336**
hsa-miR-128-3p	Undetected	
hsa-miR-130a-3p	44.819	0.0542
hsa-miR-130b-3p	46.322	0.0681
hsa-miR-132-3p	83.506	0.1379
hsa-miR-133a-3p	Undetected	
hsa-miR-133b	Undetected	
hsa-miR-136-3p	Undetected	
hsa-miR-136-5p	Undetected	
hsa-miR-139-5p	0.373	0.1388
hsa-miR-140-3p	1.292	0.7052
**hsa-miR-140-5p**	**0.016**	**0.0021**
hsa-miR-141-3p	Undetected	
hsa-miR-142-3p	0.081	0.1333
hsa-miR-142-5p	0.456	0.1198
hsa-miR-143-3p	Undetected	
hsa-miR-144-3p	0.077	0.3523
hsa-miR-144-5p	47.871	0.0885
hsa-miR-145-5p	0.501	0.1040
hsa-miR-146a-5p	0.859	0.7440
hsa-miR-146b-5p	0.821	0.4490
hsa-miR-148a-3p	0.336	0.3043
**hsa-miR-148b-3p**	**32.664**	**0.0134**

hsa-miR-150-5p	0.998	0.9955
hsa-miR-151a-3p	2.649	0.4233
hsa-miR-151a-5p	0.355	0.2331
hsa-miR-152-3p	0.670	0.5150
hsa-miR-154-5p	undetected	
**hsa-miR-155-5p**	**32.980**	**0.0003**
hsa-miR-15a-5p	1.045	0.9654
hsa-miR-15b-3p	0.756	0.3797
hsa-miR-15b-5p	2.689	0.4935
**hsa-miR-16-2-3p**	**34.059**	**0.028**
hsa-miR-16-5p	1.246	0.5375
hsa-miR-17-5p	4.460	0.5445
**hsa-miR-181a-5p**	0.015	0.0002
hsa-miR-185-5p	0.308	0.1002
**hsa-miR-186-5p**	**33.453**	**0.0285**
hsa-miR-18a-5p	0.813	0.6658
hsa-miR-18b-5p	0.315	0.2836
hsa-miR-191-5p	1.512	0.5773
hsa-miR-192-5p	0.623	0.2625
hsa-miR-193a-5p	Undetected	
hsa-miR-194-5p	0.934	0.7713
hsa-miR-195-5p	Undetected	
hsa-miR-197-3p	1.382	0.3775
hsa-miR-199a-3p	0.831	0.4247
**hsa-miR-199a-5p**	**0.008**	**0.0216**
hsa-miR-19a-3p	0.355	0.2259
hsa-miR-19b-3p	0.287	0.1947
hsa-miR-200a-3p	Undetected	
hsa-miR-200c-3p	53.405	0.2469
hsa-miR-205-5p	Undetected	
hsa-miR-208a-3p	Undetected	
hsa-miR-20a-5p	0.672	0.5256
**hsa-miR-20b-5p**	**33.930**	**0.0373**
hsa-miR-210-3p	Undetected	
hsa-miR-2110	46.391	0.0811
hsa-miR-215-5p	0.371	0.1272
hsa-miR-21-5p	0.969	0.8960
hsa-miR-221-3p	0.220	0.1396
hsa-miR-222-3p	0.699	0.4092
hsa-miR-223-3p	1.005	0.9899
hsa-miR-223-5p	0.567	0.2899
hsa-miR-22-3p	0.383	0.1559
hsa-miR-22-5p	Undetected	
hsa-miR-23a-3p	0.994	0.5644
hsa-miR-23b-3p	1.674	0.4277
hsa-miR-24-3p	1.329	0.4187
hsa-miR-25-3p	1.311	0.4434
hsa-miR-26a-5p	1.436	0.7172
hsa-miR-26b-5p	0.496	0.1636
hsa-miR-27a-3p	3.069	0.2764
**hsa-miR-27b-3p**	**0.409**	**0.0146**
**hsa-miR-28-3p**	**33.374**	**0.0011**
hsa-miR-28-5p	0.542	0.0023
**hsa-miR-29a-3p**	**0.015**	**0.0006**
**hsa-miR-29b-3p**	**34.476**	**0.0048**
hsa-miR-29c-3p	66.131	0.1851

hsa-miR-301a-3p	0.184	0.0905
hsa-miR-30a-5p	undetected	
hsa-miR-30b-5p	0.291	0.1936
hsa-miR-30c-5p	0.188	0.0830
hsa-miR-30d-5p	1.656	0.1800
**hsa-miR-30e-3p**	**32.977**	**0.0004**
hsa-miR-30e-5p	0.377	0.1258
**hsa-miR-320a**	**2.378**	**0.0183**
**hsa-miR-320b**	**3.037**	**0.0128**
hsa-miR-320c	3.268	0.2028
hsa-miR-324-3p	0.561	0.0861
hsa-miR-324-5p	52.154	0.2389
hsa-miR-32-5p	0.875	0.7812
**hsa-miR-326**	**34.710**	**0.0027**
hsa-miR-328-3p	1.154	0.8681
hsa-miR-331-3p	0.010	0.2031
**hsa-miR-335-3p**	**33.395**	**0.0274**
hsa-miR-335-5p	Undetected	
hsa-miR-338-3p	Undetected	
hsa-miR-339-3p	Undetected	
hsa-miR-339-5p	0.534	0.1940
hsa-miR-33a-5p	Undetected	
hsa-miR-342-3p	0.541	0.3048
hsa-miR-34a-5p	Undetected	
hsa-miR-361-5p	79.726	0.1168
hsa-miR-362-3p	Undetected	
hsa-miR-363-3p	0.725	0.3578
hsa-miR-365a-3p	Undetected	
hsa-miR-374a-5p	47.266	0.0692
hsa-miR-374b-5p	0.848	0.4592
hsa-miR-375	0.326	0.2856
**hsa-miR-376a-3p**	**32.728**	**0.0146**
hsa-miR-376c-3p	44.991	0.1828
**hsa-miR-378a-3p**	**0.031**	**0.0137**
**hsa-miR-382-5p**	**33.174**	**0.0010**
**hsa-miR-409-3p**	**35.541**	**0.0005**
**hsa-miR-421**	**32.351**	**0.0071**
**hsa-miR-423-3p**	**33.454**	**0.0285**
hsa-miR-423-5p	1.582	0.2810
**hsa-miR-424-5p**	**0.031**	**0.0154**
hsa-miR-425-3p	45.356	0.0771
hsa-miR-425-5p	1.364	0.5897
hsa-miR-451a	0.213	0.3515
hsa-miR-454-3p	45.439	0.0577
hsa-miR-483-5p	0.678	0.4665
hsa-miR-484	0.491	0.1685
hsa-miR-485-3p	Undetected	
hsa-miR-486-5p	4.640	0.1726
**hsa-miR-495-3p**	**32.963**	**0.0192**
hsa-miR-497-5p	Undetected	
hsa-miR-501-3p	Undetected	
**hsa-miR-502-3p**	**32.979**	**0.0195**
**hsa-miR-505-3p**	**33.851**	**0.0358**
hsa-miR-532-3p	Undetected	
hsa-miR-532-5p	0.552	0.0264
hsa-miR-543	Undetected	

**hsa-miR-574-3p**	**0.256**	**0.0016**
hsa-miR-584-5p	51.916	0.0511
hsa-miR-590-5p	0.675	0.1974
**hsa-miR-629-5p**	**34.614**	**0.0011**
hsa-miR-652-3p	0.333	0.2859
hsa-miR-660-5p	51.569	0.2351
hsa-miR-7-1-3p	Undetected	
hsa-miR-7-5p	Undetected	
hsa-miR-766-3p	0.405	0.1117
**hsa-miR-874-3p**	**32.953**	**0.0190**
hsa-miR-877-5p	46.335	0.0783
hsa-miR-885-5p	Undetected	
hsa-miR-92a-3p	4.977	0.2210
hsa-miR-92b-3p	Undetected	
**hsa-miR-93-3p**	**32.869**	**0.0173**
hsa-miR-93-5p	0.570	0.3757
hsa-miR-99a-5p	Undetected	
hsa-miR-99b-5p	0.486	0.1793
