# Supplementary material for: A Physically Active Status Affects the Circulating Profile of Cancer-Associated miRNAs
Source: Diagnostics (Basel). 2021 Apr 30;11(5):820. doi: 10.3390/diagnostics11050820 (PMC8147229; doi:10.3390/diagnostics11050820)
Supplement: Supplementary file 1 [file diagnostics-11-00820-s001.zip › Supplementary Table S2.pdf]

diagnostics

MDPI

Article

A Physically Active Status Affects the Circulating Profile of Cancer-Associated miRNAs

Martina Faraldi [1], Laura Gerosa [2,*], Marta Gomarasca [1], Veronica Sansoni [1], Silvia Perego [1], Ewa Ziemann [3], Giuseppe Banfi [1,4] and Giovanni Lombardi [1,3]

[1] IRCCS Istituto Ortopedico Galeazzi, Laboratory of Experimental Biochemistry & Molecular Biology, 20161 Milano, Italy; martina.faraldi@grupposandonato.it (M.F.); marta.gomarasca@grupposandonato.it (M.G.); veronica.sansoni@grupposandonato.it (V.S.); silvia.perego@grupposandonato.it (S.P.); banfi.giuseppe@unisr.it (G.B.); giovanni.lombardi@grupposandonato.it (G.L.)

[2] Gruppo San Donato Foundation, 20122 Milano, Italy

[3] Department of Athletics, Strength and Conditioning, Poznań University of Physical Education, 61-871 Poznań, Poland; ziemann@awf.poznan.pl

[4] Vita-Salute San Raffaele University, 20132 Milano, Italy

* Correspondence: laura.gerosa@grupposandonato.it; Tel.: +39-02166214068

Citation: Faraldi, M.; Gerosa, L.; Gomarasca, M.; Sansoni, V.; Perego, S.; Ziemann, E.; Banfi, G.; Lombardi, G. A Physically Active Status Affects the Circulating Profile of Cancer-Associated miRNAs. *Diagnostics* **2021**, *11*, 820. https://doi.org/10.3390/diagnostics11050820

Academic Editor: Anastasia Malek

Received: 24 March 2021
Accepted: 29 April 2021
Published: date

Publisher's Note: MDPI stays neutral with regard to jurisdictional claims in published maps and institutional affiliations.

Copyright: © 2021 by the authors. Licensee MDPI, Basel, Switzerland. This article is an open access article distributed under the terms and conditions of the Creative Commons Attribution (CC BY) license (http://creativecommons.org/licenses/by/4.0/).

Table S2. List of the miRNAs and miRNA signatures associated with diagnosis in Liver Cancer from literature. Only miRNAs with significant clinically relevance (AUC≥0.7), and signature with at least one modulated miRNA from our experimental setting were considered.

Cancer	Sample	miRNAs	miRNA signatures	Regulation	AUC	Reference
LA	Serum	miR-210-3p		miR-210-3p ↑	miR-210-3p: 0.840	[1]
LA	Serum exosomes	miR-126-3p<br>miR-140-5p<br>miR-21-5p		miR-126-3p ↑<br>miR-140-5p ↑<br>miR-21-5p ↑	miR-126-3p: 0.910<br>miR-140-5p: 0.880<br>miR-21-5p: 0.970	[2]
LA	Serum	miR-30c-1*		miR-30c-1* ↑	miR-30c-1*: 0.740	[3]
LA	Serum	miR-142-5p<br>miR-146a-5p<br>miR-223-3p<br>miR-409-3p	miR-142-5p<br>miR-146a-5p<br>miR-223-3p<br>miR-409-3p	miR-142-5p ↑<br>miR-146a-5p ↓<br>miR-223-3p ↑<br>miR-409-3p ↓	miR-142-5p: 0.743<br>miR-146a-5p: 0.745<br>miR-223-3p: 0828<br>miR-409-3p: 0.755<br>signature: 0.933	[4]
LC	Serum	miR-125b<br>miR-21		miR-125b ↑<br>miR-21 ↑	miR-125b: 0.877<br>miR-21: 0.821	[5]
LC	Serum	miR-154-5p		miR-154-5p ↓	miR-154-5p: 0.957	[6]
LC	Serum	miR-155		miR-155 ↑	miR-155: 0.761	[7]
LC	Serum	miR-92a	let-7d<br>miR-140-5p<br>miR-148a<br>miR-191<br>miR-223<br>miR-29a<br>miR-30b<br>miR-30c<br>miR-328<br>miR-331-3p<br>miR-374a<br>miR-484<br>miR-92a	let-7d ↑<br>miR-140-5p ↑<br>miR-148a ↑<br>miR-191 ↑<br>miR-223 ↑<br>miR-29a ↑<br>miR-30b ↑<br>miR-30c ↑<br>miR-328 ↑<br>miR-331-3p ↑<br>miR-374a ↑<br>miR-484 ↑<br>miR-92a ↑	miR-92a: 0.781<br>signature miR-92a, miR-140-5p: 0.923<br>signature miR-92a, miR-140-5p, miR-331-3p: 0.969<br>signature miR-92a, miR-140-5p, miR-331-3p, miR-374a: 0.970<br>signature miR-92a, miR-140-5p, miR-331-3p, miR-374a, miR-223: 0.986<br>signature miR-92a, miR-140-5p, miR-331-3p, miR-374a, miR-223, miR-148a: 0.987<br>signature miR-92a, miR-140-5p, miR-331-3p, miR-374a, miR-223, miR-148a, miR-484: 0.988<br>signature miR-92a, miR-140-5p, miR-331-3p, miR-374a, miR-223, miR-148a, miR-484, miR-328: 0.989<br>signature miR-92a, miR-140-5p, miR-331-3p, miR-374a, miR-223, miR-148a, miR-484, miR-328, miR-191: 0.990<br>signature miR-92a, miR-140-5p, miR-331-3p, miR-374a, miR-223, miR-148a, miR-484, miR-328, miR-191, miR-30c: 0.996<br>signature miR-92a, miR-140-5p, miR-331-3p, miR-374a, miR-223, miR-148a, miR-484, miR-328, miR-191, miR-30c, miR-29a: 0.996<br>signature miR-92a, miR-140-5p, miR-331-3p, miR-374a, miR-223, miR-148a, miR-484, miR-328, miR-191, miR-30c, miR-29a, let-7d: 0.997<br>signature miR-92a, miR-140-5p, miR-331-3p, miR-374a, miR-223, miR-148a, miR-484, miR-328, miR-191, miR-30c, miR-29a, let-7d, miR-30b: 0.996	[8]
NSCLC	Serum	miR-17<br>miR-18a<br>miR-20a<br>miR-92a			Normal subjects vs. stage I-III patients AUC:<br>miR-17: 0.810<br>miR-18a: 0.739<br>miR-20a: 0.897<br>miR-92a: 0.810<br>Normal subjects vs. stage IV patients AUC:<br>miR-17: 1.000<br>miR-18a: 0.991<br>miR-20a: 1.000<br>miR-92a: 0.834	[9]
NSCLC	Serum	miR-30		miR-30 ↓	miR-30: 0.802	[10]
NSCLC	Serum	miR-233		miR-233 ↑	miR-233: 0.808	[11]
NSCLC	Serum exosomes	miR-17-5p		miR-17-5p ↑	miR-17-5p: 0.738	[12]
NSCLC	Serum	let-7a<br>miR-21	let-7a<br>miR-21	let-7a ↓<br>miR-21 ↑	let-7a: 0.711<br>miR-21: 0.831<br>signature: 0.879	[13]
NSCLC	Serum exosomes	miR-126		miR-126 ↑	miR-126: 0.859	[14]
NSCLC	Serum	miR-126<br>miR-22		miR-126 ↓<br>miR-22 ↑	miR-126: 0.874<br>miR-22: 0.916	[15]
NSCLC	Serum	miR-125b<br>miR-22		miR-125b ↑<br>miR-22 ↑	miR-125b: 0.704<br>miR-22: 0.725	[16]
NSCLC	Serum	miR-125b<br>miR-205		miR-125b ↑<br>miR-205 ↑	miR-125b: 0.765<br>miR-205: 0.800	[17]
NSCLC	Serum	miR-126		miR-126 ↑	miR-126: 0.793	[18]
NSCLC	Serum	miR-21		miR-21 ↑	miR-21: 0.918	[19]

NSCLC	Serum	miR-15b-5p<br>miR-16-5p<br>miR-20a-5p	miR-15b-5p<br>miR-16-5p<br>miR-20a-5p	miR-15b-5p ↑<br>miR-16-5p ↓<br>miR-20a-5p ↓	miR-15b-5p: >0.700<br>miR-16-5p: >0.700<br>miR-20a-5p: >0.700<br>signature: 0.930	[20]
NSCLC	Serum	miR-25<br>miR-483-5p<br>miR-7		miR-25 ↑<br>miR-483-5p ↑<br>miR-7 ↑	Chinese cohort:<br>miR-25: 0.835<br>miR-7 0.806<br>American cohort:<br>miR-483-5p: 0.932<br>miR-7: 0.964	[21]
NSCLC	Serum	let-7g<br>miR-106a<br>miR-106b<br>miR-141<br>miR-17<br>miR-19a<br>miR-19b<br>miR-200c<br>miR-20b<br>miR-215<br>miR-24<br>miR-25<br>miR-301<br>miR-331<br>miR-93		let-7g ↑<br>miR-106a ↑<br>miR-106b ↑<br>miR-141 ↑<br>miR-17 ↑<br>miR-19a ↑<br>miR-19b ↑<br>miR-200c ↑<br>miR-20b ↑<br>miR-215 ↑<br>miR-24 ↑<br>miR-25 ↑<br>miR-301 ↑<br>miR-331 ↑<br>miR-93 ↑	let-7g: 0.840<br>miR-106a: 0.958<br>miR-106b: 0.838<br>miR-141: 0.875<br>miR-17: 0.918<br>miR-19a: 0.948<br>miR-19b: 0.916<br>miR-200c: 0.820<br>miR-20b: 0.875<br>miR-215: 0.828<br>miR-24: 0.715<br>miR-25: 0.826<br>miR-301: 0.841<br>miR-331: 0.748<br>miR-93: 0.899	[22]
NSCLC	Serum	miR-125a-5p<br>miR-145<br>miR-146a		miR-125a-5p ↑<br>miR-145 ↑<br>miR-146a ↑	miR-125a-5p: 0.710<br>miR-145: 0.840<br>miR-146a: 0.780	[23]
NSCLC	Serum	miR-125a-5p<br>miR-126<br>miR-25	miR-125a-5p<br>miR-126<br>miR-25	miR-125a-5p ↓<br>miR-126 ↓<br>miR-25 ↓	miR-125a-5p: 0.833<br>miR-126: 0.786<br>miR-25: 0.818<br>signature: 0.936	[24]
NSCLC	Serum	miR-148a<br>miR-148b<br>miR-152<br>miR-21	miR-148a<br>miR-148b<br>miR-152<br>miR-21	miR-148a ↓<br>miR-148b ↓<br>miR-152 ↓<br>miR-21 ↑	miR-148a: 0.900<br>miR-148b: 0.900<br>miR-152: 0.820<br>miR-21: 0.810<br>signature: 0.970	[25]
NSCLC	Serum	miR-145<br>miR-152<br>miR-199a-5p<br>miR-20a<br>miR-221<br>miR-222<br>miR-223<br>miR-24<br>miR-25<br>miR-320	miR-145<br>miR-152<br>miR-199a-5p<br>miR-20a<br>miR-221<br>miR-222<br>miR-223<br>miR-24<br>miR-25<br>miR-320	miR-145 ↑<br>miR-152 ↑<br>miR-199a-5p ↑<br>miR-20a ↑<br>miR-221 ↑<br>miR-222 ↑<br>miR-223 ↑<br>miR-24 ↑<br>miR-25 ↑<br>miR-320 ↑	miR-145: > 0.800<br>miR-152: > 0.800<br>miR-199a-5p: > 0.800<br>miR-20a: > 0.800<br>miR-221: > 0.800<br>miR-222: > 0.800<br>miR-223: > 0.800<br>miR-24: > 0.800<br>miR-25: > 0.800<br>miR-320: > 0.800<br>signature: 0.972	[26]
NSCLC	Serum	miR-29c		miR-29c ↑	miR-29c: 0.723	[27]
NSCLC	Serum	miR-125b		miR-125b ↓	miR-125b: 0.786	[28]
LC	Serum	miR-146b<br>miR-205<br>miR-29c<br>miR-30b	miR-146b<br>miR-205<br>miR-29c<br>miR-30b	miR-146b ↑<br>miR-205 ↑<br>miR-29c ↑<br>miR-30b ↑	miR-146b: 0.850<br>miR-205: 0.920<br>miR-29c: 0.890<br>miR-30b: 0.76<br>signature: 0.930	[29]
LA	Serum				miR-146b: 0.930<br>miR-205: 0.930<br>miR-29c: 0.980<br>miR-30b: 0.870<br>signature: 0.980	
LSCC	Serum				miR-146b: 0.770<br>miR-205: 0.880<br>miR-29c: 0.830<br>signature: 0.930	
NSCLC stage I	Serum				miR-146b: 0.840<br>miR-205: 0.900<br>miR-29c: 0.910<br>miR-30b: 0.710<br>signature: 0.960	
NSCLC stage II	Serum				miR-146b: 0.880<br>miR-205: 0.910	

					miR-29c: 0.960<br>miR-30b: 0.780<br>signature: 0.950	
LA	Plasma extracellular vesicles	miR-194-5p<br>miR-451a<br>miR-486-5p	miR-194-5p<br>miR-451a<br>miR-486-5p	miR-194-5p ↑<br>miR-451a ↑<br>miR-486-5p ↑	miR-194-5p: 0.749<br>miR-451a: 0.904<br>miR-486-5p: 0.957<br>Signature: 0.965	[30]
LA	Plasma	miR-21<br>miR-339-5p	miR-21<br>miR-339-5p	miR-21 ↑<br>miR-339-5p ↑	miR-21: 0.880<br>miR-339-5p: 0.900<br>signature: 0.963	[31]
LA	Plasma		miR-19b-3p<br>miR-21-5p<br>miR-221-3p<br>miR-409-3p<br>miR-425-5p<br>miR-584-5p	miR-19b-3p ↑<br>miR-21-5p ↑<br>miR-221-3p ↑<br>miR-409-3p ↑<br>miR-425-5p ↑<br>miR-584-5p ↑	signature: 0.740	[32]
LC	Plasma	miR-30a-5p		miR-30a-5p ↑	miR-30a-5p: 0.820	[33]
LC	Plasma	miR-21		miR-21↑	miR-21: 0.891	[34]
LC	Plasma	miR-145<br>miR-155<br>miR-21	miR-145<br>miR-155<br>miR-21	miR-145 ↓<br>miR-155 ↑<br>miR-21 ↑	miR-21: 0.709<br>miR-155: 0.740<br>signature miR-145, miR-21: 0.847<br>signature miR-145, miR-155: 0.833<br>signature miR-155, mIrR-21: 0.727<br>signature miR-145, miR-155, miR-21: 0.872	[35]
LC	Plasma	miR-155<br>miR-197		miR-155 ↑<br>miR-197 ↑	miR-155: 0.865<br>miR-197: 0.879	[36]
LSCC	Plasma	miR-106a-5p<br>miR-181a-5p<br>miR-21-5p	miR-106a-5p<br>miR-181a-5p<br>miR-21-5p<br>miR-93-5p	miR-106a-5p ↑<br>miR-181a-5p ↑<br>miR-21-5p ↑<br>miR-93-5p ↑	miR-106a-5p: 0.737<br>miR-181a-5p: 0.731<br>miR-21-5p: 0.739<br>signature: 0.827	[37]
LSCC	Plasma	miR-324-3p		miR-324-3p ↑	miR-324-3p: 0.790	[38]
NSCLC	Plasma		miR-23a<br>miR-451	miR-23a ↑<br>miR-451 ↓	signature: 0.900	[39]
NSCLC	Plasma	miR-126-3p<br>miR-145<br>miR-205-5p	miR-126-3p<br>miR-145<br>miR-205-5p<br>miR-210-3p		miR-126-3p: 0.768<br>miR-145: 0.713<br>miR-205-5p: 0.732<br>signature: 0.963	[40]
NSCLC	Plasma	miR-25		miR-25 ↑	miR-25: 0.832	[41]
NSCLC	Plasma	miR-17		miR-17 ↑	miR-17: 0.833	[42]
NSCLC	plasma	miR-23a		miR-23a ↑	miR-23a: 0.742	[43]
NSCLC	Plasma	miR-16<br>miR-486	miR-16<br>miR-205<br>miR-486	miR-16 ↑<br>miR-205 ↑<br>miR-486 ↑	miR-16: >0.700<br>miR-486: >0.700<br>signature: 0.890	[44]
NSCLC	Plasma exosomes		let-7b-5p<br>let-7e-5p<br>miR-21-5p<br>miR-24-3p	let-7b-5p ↑<br>let-7e-5p ↓<br>miR-21-5p ↓<br>miR-24-3p ↑	signature: 0.899	[45]
NSCLC	Plasma	miR-339-3p<br>miR-425-3p		miR-339-3p ↑<br>miR-425-3p ↑	miR-339-3p: 0.723<br>miR-425-3p: 0.738	[46]
NSCLC	Plasma	miR-30a		miR-30a ↑	miR-30a: 0.727	[47]
NSCLC	Plasma	miR-150<br>miR-486		miR-150 ↑<br>miR-486 ↑	miR-150: 0.752<br>miR-486: 0.926	[48]
NSCLC	Plasma	miR-145<br>miR-155<br>miR-20a<br>miR-21<br>miR-223		miR-145 ↑<br>miR-155 ↑<br>miR-20a ↑<br>miR-21 ↑<br>miR-223 ↑	miR-145: 0.770<br>miR-155: 0.920<br>miR-20a: 0.890<br>miR-21: 0.770<br>miR-223: 0.940	[49]
NSCLC	Plasma	miR-21		miR-21 ↑	miR-21: 0.775	[50]
NSCLC	Plasma	miR-126<br>miR-210<br>miR-21<br>miR-486-5p	miR-126<br>miR-210<br>miR-21<br>miR-486-5p	miR-126 ↓<br>miR-210 ↑<br>miR-21 ↑<br>miR-486-5p ↓	miR-126: 0.760<br>miR-210: 0.746<br>miR-21: 0.816<br>miR-486-5p: 0.878<br>signature: 0.926	[51]
NSCLC	Plasma				miR-195: 0.820<br>miR-27-3p: 0.950	
LC	Plasma	miR-195<br>miR-27a-3p		miR-195 ↓<br>miR-27-3p ↑	miR-195: 0.760<br>miR-27a-3p: 0.900	[52]
SCLC	Plasma				miR-195: 0.710<br>miR-27a-3p: 0.900	
LA	Plasma	miR-126a-5p<br>miR-139-5p		miR-126a-5p ↑<br>miR-139-5p ↑	miR-126a-5p: 0.798<br>miR-139-5p: 0.969	[53]

		miR-152-3p	miR-152-3p ↑	miR-152-3p: 0.941	
		miR-200c-3p	miR-200c-3p ↑	miR-200c-3p: 0.948	
		miR-26a-5p	miR-26a-5p ↑	miR-26a-5p: 0.757	
		miR-451a	miR-451a ↓	miR-451a: 0.7971	
LSCC	Plasma	miR-126-5p	miR-126-5p ↑	miR-126-5p: 0.957	
		miR-139-5p	miR-139-5p ↑	miR-139-5p: 0.992	
		miR-151a-5p	miR-151a-5p ↑	miR-151a-5p: 0.953	
		miR-151a-3p	miR-151a-3p ↑	miR-151a-3p: 0.915	
		miR-152-3p	miR-152-3p ↑	miR-152-3p: 0.991	
		miR-26a-5p	miR-26a-5p ↑	miR-26a-5p: 0.917	
LA+LSCC	Plasma	miR-126-5p	miR-126-5p ↑	miR-126-5p: 0.880	
		miR-139-5p	miR-139-5p ↑	miR-139-5p: 0.980	
		miR-152-3p	miR-152-3p ↑	miR-152-3p: 0.965	
		miR-26a-5p	miR-26a-5p ↑	miR-26a-5p: 0.834	
NSCLC	Plasma			miR-141: 0.972	
LA	Plasma	miR-141	miR-141 ↑	miR-141: 0.969	[54]
LSCC	Plasma			miR-141: 0.976	
		miR-148a-3p	miR-148a-3p ↓	miR-148a-3p: 0.826	
NSCLC	Blood	miR-152	miR-152 ↓	miR-152: 0.757	[55]
		miR-21-5p	miR-21-5p↑	miR-21-5p: 0.850	

LA: lung adenocarcinoma; LC: lung cancer; NSCLC: non-small cell lung cancer; SCLC small cell lung carcinoma; LSCC: lung squamous cell carcinoma; AUC: area under the ROC curve.

References

1. Switlik, W.Z.; Karbownik, M.S.; Suwalski, M.; Kozak, J.; Szemraj, J. Serum miR-210-3p as a Potential Noninvasive Biomarker of Lung Adenocarcinoma: A Preliminary Study. *Genetic testing and molecular biomarkers* **2019**, *23*, 353-358, doi:10.1089/gtmb.2018.0275.
2. Feng, M.; Zhao, J.; Wang, L.; Liu, J. Upregulated Expression of Serum Exosomal microRNAs as Diagnostic Biomarkers of Lung Adenocarcinoma. *Annals of clinical and laboratory science* **2018**, *48*, 712-718.
3. Rani, S.; Gately, K.; Crown, J.; O'Byrne, K.; O'Driscoll, L. Global analysis of serum microRNAs as potential biomarkers for lung adenocarcinoma. *Cancer biology & therapy* **2013**, *14*, 1104-1112, doi:10.4161/cbt.26370.
4. Wang, J.; Zhang, C.; Peng, X.; Liu, K.; Zhao, L.; Chen, X.; Yu, H.; Lai, Y. A combination of four serum miRNAs for screening of lung adenocarcinoma. *Human cell* **2020**, *33*, 830-838, doi:10.1007/s13577-020-00346-6.
5. Pastor-Navarro, B.; Garcia-Flores, M.; Fernandez-Serra, A.; Blanch-Tormo, S.; Martinez de Juan, F.; Martinez-Lapiedra, C.; Maia de Alcantara, F.; Penalver, J.C.; Cervera-Deval, J.; Rubio-Briones, J.; et al. A Tetra-Panel of Serum Circulating miRNAs for the Diagnosis of the Four Most Prevalent Tumor Types. *International journal of molecular sciences* **2020**, *21*, doi:10.3390/ijms21082783.
6. Huang, J.; Wu, J.; Li, Y.; Li, X.; Yang, T.; Yang, Q.; Jiang, Y. Deregulation of serum microRNA expression is associated with cigarette smoking and lung cancer. *BioMed research international* **2014**, *2014*, 364316, doi:10.1155/2014/364316.
7. Gao, F.; Chang, J.; Wang, H.; Zhang, G. Potential diagnostic value of miR-155 in serum from lung adenocarcinoma patients. *Oncology reports* **2014**, *31*, 351-357, doi:10.3892/or.2013.2830.
8. Zhang, Y.H.; Jin, M.; Li, J.; Kong, X. Identifying circulating miRNA biomarkers for early diagnosis and monitoring of lung cancer. *Biochimica et biophysica acta. Molecular basis of disease* **2020**, *1866*, 165847, doi:10.1016/j.bbadis.2020.165847.
9. Yang, C.; Jia, X.; Zhou, J.; Sun, Q.; Ma, Z. The MiR-17-92 Gene Cluster is a Blood-Based Marker for Cancer Detection in Non-Small-Cell Lung Cancer. *The American journal of the medical sciences* **2020**, *360*, 248-260, doi:10.1016/j.amjms.2020.05.004.
10. Sui, J. MiRNA-30 Play an Important Role in Non-Small Cell Lung Cancer (NSCLC). *Clinical laboratory* **2020**, *66*, doi:10.7754/Clin.Lab.2019.190712.
11. D'Antona, P.; Cattoni, M.; Dominioni, L.; Poli, A.; Moretti, F.; Cinquetti, R.; Gini, E.; Daffre, E.; Noonan, D.M.; Imperatori, A.; et al. Serum miR-223: A Validated Biomarker for Detection of Early-Stage Non-Small Cell Lung Cancer. *Cancer epidemiology, biomarkers & prevention : a publication of the American Association for Cancer Research, cosponsored by the American Society of Preventive Oncology* **2019**, *28*, 1926-1933, doi:10.1158/1055-9965.EPI-19-0626.
12. Zhang, Y.; Zhang, Y.; Yin, Y.; Li, S. Detection of circulating exosomal miR-17-5p serves as a novel non-invasive diagnostic marker for non-small cell lung cancer patients. *Pathology, research and practice* **2019**, *215*, 152466, doi:10.1016/j.prp.2019.152466.
13. Wang, X.; Jia, Z.; Shi, H.; Pan, C. Identification and evaluation of 2 circulating microRNAs for non-small cell lung cancer diagnosis. *Clinical and experimental pharmacology & physiology* **2018**, *45*, 1083-1086, doi:10.1111/1440-1681.12977.
14. Grimolizzi, F.; Monaco, F.; Leoni, F.; Bracci, M.; Staffolani, S.; Bersaglieri, C.; Gaetani, S.; Valentino, M.; Amati, M.; Rubini, C.; et al. Exosomal miR-126 as a circulating biomarker in non-small-cell lung cancer regulating cancer progression. *Scientific reports* **2017**, *7*, 15277, doi:10.1038/s41598-017-15475-6.
15. Shang, A.Q.; Xie, Y.N.; Wang, J.; Sun, L.; Wei, J.; Lu, W.Y.; Lan, J.Y.; Wang, W.W.; Wang, L.; Wang, L.L. Predicative values of serum microRNA-22 and microRNA-126 levels for non-small cell lung cancer development and metastasis: a case-control study. *Neoplasma* **2017**, *64*, 453-459, doi:10.4149/neo_2017_317.
16. Shi, G.L.; Chen, Y.; Sun, Y.; Yin, Y.J.; Song, C.X. Significance of Serum MicroRNAs in the Auxiliary Diagnosis of Non-Small Cell Lung Cancer. *Clinical laboratory* **2017**, *63*, 133-140, doi:10.7754/Clin.Lab.2016.160710.

17. Halvorsen, A.R.; Bjaanaes, M.; LeBlanc, M.; Holm, A.M.; Bolstad, N.; Rubio, L.; Penalver, J.C.; Cervera, J.; Mojarrieta, J.C.; Lopez-Guerrero, J.A.; et al. A unique set of 6 circulating microRNAs for early detection of non-small cell lung cancer. *Oncotarget* **2016**, *7*, 37250-37259, doi:10.18632/oncotarget.9363.
18. Zhu, W.; Zhou, K.; Zha, Y.; Chen, D.; He, J.; Ma, H.; Liu, X.; Le, H.; Zhang, Y. Diagnostic Value of Serum miR-182, miR-183, miR-210, and miR-126 Levels in Patients with Early-Stage Non-Small Cell Lung Cancer. *PloS one* **2016**, *11*, e0153046, doi:10.1371/journal.pone.0153046.
19. Sun, M.; Song, J.; Zhou, Z.; Zhu, R.; Jin, H.; Ji, Y.; Lu, Q.; Ju, H. Comparison of Serum MicroRNA21 and Tumor Markers in Diagnosis of Early Non-Small Cell Lung Cancer. *Disease markers* **2016**, *2016*, 3823121, doi:10.1155/2016/3823121.
20. Fan, L.; Qi, H.; Teng, J.; Su, B.; Chen, H.; Wang, C.; Xia, Q. Identification of serum miRNAs by nano-quantum dots microarray as diagnostic biomarkers for early detection of non-small cell lung cancer. *Tumour biology : the journal of the International Society for Oncodevelopmental Biology and Medicine* **2016**, *37*, 7777-7784, doi:10.1007/s13277-015-4608-3.
21. Wang, C.; Ding, M.; Xia, M.; Chen, S.; Van Le, A.; Soto-Gil, R.; Shen, Y.; Wang, N.; Wang, J.; Gu, W.; et al. A Five-miRNA Panel Identified From a Multicentric Case-control Study Serves as a Novel Diagnostic Tool for Ethnically Diverse Non-small-cell Lung Cancer Patients. *EBioMedicine* **2015**, *2*, 1377-1385, doi:10.1016/j.ebiom.2015.07.034.
22. Nadal, E.; Truini, A.; Nakata, A.; Lin, J.; Reddy, R.M.; Chang, A.C.; Ramnath, N.; Gotoh, N.; Beer, D.G.; Chen, G. A Novel Serum 4-microRNA Signature for Lung Cancer Detection. *Scientific reports* **2015**, *5*, 12464, doi:10.1038/srep12464.
23. Wang, R.J.; Zheng, Y.H.; Wang, P.; Zhang, J.Z. Serum miR-125a-5p, miR-145 and miR-146a as diagnostic biomarkers in non-small cell lung cancer. *International journal of clinical and experimental pathology* **2015**, *8*, 765-771.
24. Wang, P.; Yang, D.; Zhang, H.; Wei, X.; Ma, T.; Cheng, Z.; Hong, Q.; Hu, J.; Zhuo, H.; Song, Y.; et al. Early Detection of Lung Cancer in Serum by a Panel of MicroRNA Biomarkers. *Clinical lung cancer* **2015**, *16*, 313-319 e311, doi:10.1016/j.cllc.2014.12.006.
25. Yang, J.S.; Li, B.J.; Lu, H.W.; Chen, Y.; Lu, C.; Zhu, R.X.; Liu, S.H.; Yi, Q.T.; Li, J.; Song, C.H. Serum miR-152, miR-148a, miR-148b, and miR-21 as novel biomarkers in non-small cell lung cancer screening. *Tumour biology : the journal of the International Society for Oncodevelopmental Biology and Medicine* **2015**, *36*, 3035-3042, doi:10.1007/s13277-014-2938-1.
26. Chen, X.; Hu, Z.; Wang, W.; Ba, Y.; Ma, L.; Zhang, C.; Wang, C.; Ren, Z.; Zhao, Y.; Wu, S.; et al. Identification of ten serum microRNAs from a genome-wide serum microRNA expression profile as novel noninvasive biomarkers for nonsmall cell lung cancer diagnosis. *International journal of cancer* **2012**, *130*, 1620-1628, doi:10.1002/ijc.26177.
27. Zhu, W.; He, J.; Chen, D.; Zhang, B.; Xu, L.; Ma, H.; Liu, X.; Zhang, Y.; Le, H. Expression of miR-29c, miR-93, and miR-429 as potential biomarkers for detection of early stage non-small lung cancer. *PloS one* **2014**, *9*, e87780, doi:10.1371/journal.pone.0087780.
28. Yuxia, M.; Zhennan, T.; Wei, Z. Circulating miR-125b is a novel biomarker for screening non-small-cell lung cancer and predicts poor prognosis. *Journal of cancer research and clinical oncology* **2012**, *138*, 2045-2050, doi:10.1007/s00432-012-1285-0.
29. Yang, X.; Zhang, Q.; Zhang, M.; Su, W.; Wang, Z.; Li, Y.; Zhang, J.; Beer, D.G.; Yang, S.; Chen, G. Serum microRNA Signature Is Capable of Early Diagnosis for Non-Small Cell Lung Cancer. *International journal of biological sciences* **2019**, *15*, 1712-1722, doi:10.7150/ijbs.33986.
30. Yao, B.; Qu, S.; Hu, R.; Gao, W.; Jin, S.; Liu, M.; Zhao, Q. A panel of miRNAs derived from plasma extracellular vesicles as novel diagnostic biomarkers of lung adenocarcinoma. *FEBS open bio* **2019**, *9*, 2149-2158, doi:10.1002/2211-5463.12753.
31. Sun, Y.; Mei, H.; Xu, C.; Tang, H.; Wei, W. Circulating microRNA-339-5p and -21 in plasma as an early detection predictors of lung adenocarcinoma. *Pathology, research and practice* **2018**, *214*, 119-125, doi:10.1016/j.prp.2017.10.011.
32. Zhou, X.; Wen, W.; Shan, X.; Zhu, W.; Xu, J.; Guo, R.; Cheng, W.; Wang, F.; Qi, L.W.; Chen, Y.; et al. A six-microRNA panel in plasma was identified as a potential biomarker for lung adenocarcinoma diagnosis. *Oncotarget* **2017**, *8*, 6513-6525, doi:10.18632/oncotarget.14311.
33. Liang, L.B.; Zhu, W.J.; Chen, X.M.; Luo, F.M. Plasma miR-30a-5p as an early novel noninvasive diagnostic and prognostic biomarker for lung cancer. *Future oncology* **2019**, *15*, 3711-3721, doi:10.2217/fon-2019-0393.
34. Abu-Duhier, F.M.; Javid, J.; Sughayer, M.A.; Mir, R.; Albalawi, T.; Alauddin, M.S. Clinical Significance of Circulatory miRNA-21 as an Efficient Non-Invasive Biomarker for the Screening of Lung Cancer Patients. *Asian Pacific journal of cancer prevention : APJCP* **2018**, *19*, 2607-2611, doi:10.22034/APJCP.2018.19.9.2607.
35. Tang, D.; Shen, Y.; Wang, M.; Yang, R.; Wang, Z.; Sui, A.; Jiao, W.; Wang, Y. Identification of plasma microRNAs as novel noninvasive biomarkers for early detection of lung cancer. *European journal of cancer prevention : the official journal of the European Cancer Prevention Organisation* **2013**, *22*, 540-548, doi:10.1097/CEJ.0b013e32835f3be9.
36. Zheng, D.; Haddadin, S.; Wang, Y.; Gu, L.Q.; Perry, M.C.; Freter, C.E.; Wang, M.X. Plasma microRNAs as novel biomarkers for early detection of lung cancer. *International journal of clinical and experimental pathology* **2011**, *4*, 575-586.
37. Shan, X.; Zhang, H.; Zhang, L.; Zhou, X.; Wang, T.; Zhang, J.; Shu, Y.; Zhu, W.; Wen, W.; Liu, P. Identification of four plasma microRNAs as potential biomarkers in the diagnosis of male lung squamous cell carcinoma patients in China. *Cancer medicine* **2018**, *7*, 2370-2381, doi:10.1002/cam4.1490.
38. Gao, X.; Wang, Y.; Zhao, H.; Wei, F.; Zhang, X.; Su, Y.; Wang, C.; Li, H.; Ren, X. Plasma miR-324-3p and miR-1285 as diagnostic and prognostic biomarkers for early stage lung squamous cell carcinoma. *Oncotarget* **2016**, *7*, 59664-59675, doi:10.18632/oncotarget.11198.
39. Cui, S.; Cao, Z.; Guo, W.; Yu, H.; Huang, R.; Wu, Y.; Zhou, Y. [Plasma miRNA-23a and miRNA-451 as candidate biomarkers for early diagnosis of nonsmall cell lung cancer: a case-control study]. *Nan fang yi ke da xue xue bao = Journal of Southern Medical University* **2019**, *39*, 705-711, doi:10.12122/j.issn.1673-4254.2019.06.12.

40. Li, J.; Fang, H.; Jiang, F.; Ning, Y. External validation of a panel of plasma microRNA biomarkers for lung cancer. *Biomarkers in medicine* **2019**, *13*, 1557-1564, doi:10.2217/bmm-2019-0213.
41. Zhang, Y.L.; Zhang, Z.L.; Zhu, X.B.; Xu, L.; Lu, P.; Xu, M.; Liu, W.J.; Zhang, X.Y.; Yao, H.M.; Ye, X.W. Low plasma miR-25 expression is a favorite prognosis factor in non-small cell lung cancer. *European review for medical and pharmacological sciences* **2019**, *23*, 5251-5259, doi:10.26355/eurrev_201906_18191.
42. Hetta, H.F.; Zahran, A.M.; El-Mahdy, R.I.; Nabil, E.E.; Esmaeel, H.M.; Elkady, O.A.; Elkady, A.; Mohareb, D.A.; Mahmoud Mostafa, M.; John, J. Assessment of Circulating miRNA-17 and miRNA-222 Expression Profiles as Non-Invasive Biomarkers in Egyptian Patients with Non-Small-Cell Lung Cancer. *Asian Pacific journal of cancer prevention : APJCP* **2019**, *20*, 1927-1933, doi:10.31557/APJCP.2019.20.6.1927.
43. Hetta, H.F.; Zahran, A.M.; Shafik, E.A.; El-Mahdy, R.I.; Mohamed, N.A.; Nabil, E.E.; Esmaeel, H.M.; Alkady, O.A.; Elkady, A.; Mohareb, D.A.; et al. Circulating miRNA-21 and miRNA-23a Expression Signature as Potential Biomarkers for Early Detection of Non-Small-Cell Lung Cancer. *MicroRNA* **2019**, *8*, 206-215, doi:10.2174/1573399815666190115151500.
44. Sromek, M.; Glogowski, M.; Chechlinska, M.; Kulinczak, M.; Szafron, L.; Zakrzewska, K.; Owczarek, J.; Wisniewski, P.; Wlodarczyk, R.; Talarek, L.; et al. Changes in plasma miR-9, miR-16, miR-205 and miR-486 levels after non-small cell lung cancer resection. *Cellular oncology* **2017**, *40*, 529-536, doi:10.1007/s13402-017-0334-8.
45. Jin, X.; Chen, Y.; Chen, H.; Fei, S.; Chen, D.; Cai, X.; Liu, L.; Lin, B.; Su, H.; Zhao, L.; et al. Evaluation of Tumor-Derived Exosomal miRNA as Potential Diagnostic Biomarkers for Early-Stage Non-Small Cell Lung Cancer Using Next-Generation Sequencing. *Clinical cancer research : an official journal of the American Association for Cancer Research* **2017**, *23*, 5311-5319, doi:10.1158/1078-0432.CCR-17-0577.
46. Wang, Y.; Zhao, H.; Gao, X.; Wei, F.; Zhang, X.; Su, Y.; Wang, C.; Li, H.; Ren, X. Identification of a three-miRNA signature as a blood-borne diagnostic marker for early diagnosis of lung adenocarcinoma. *Oncotarget* **2016**, *7*, 26070-26086, doi:10.18632/oncotarget.8429.
47. Sun, L.; Chen, Y.; Su, Q.; Tang, X.; Liang, Y.; Che, G.; Luo, F. Increased Plasma miRNA-30a as a Biomarker for Non-Small Cell Lung Cancer. *Medical science monitor : international medical journal of experimental and clinical research* **2016**, *22*, 647-655, doi:10.12659/msm.897330.
48. Li, W.; Wang, Y.; Zhang, Q.; Tang, L.; Liu, X.; Dai, Y.; Xiao, L.; Huang, S.; Chen, L.; Guo, Z.; et al. MicroRNA-486 as a Biomarker for Early Diagnosis and Recurrence of Non-Small Cell Lung Cancer. *PloS one* **2015**, *10*, e0134220, doi:10.1371/journal.pone.0134220.
49. Geng, Q.; Fan, T.; Zhang, B.; Wang, W.; Xu, Y.; Hu, H. Five microRNAs in plasma as novel biomarkers for screening of early-stage non-small cell lung cancer. *Respiratory research* **2014**, *15*, 149, doi:10.1186/s12931-014-0149-3.
50. Wei, J.; Gao, W.; Zhu, C.J.; Liu, Y.Q.; Mei, Z.; Cheng, T.; Shu, Y.Q. Identification of plasma microRNA-21 as a biomarker for early detection and chemosensitivity of non-small cell lung cancer. *Chinese journal of cancer* **2011**, *30*, 407-414, doi:10.5732/cjc.010.10522.
51. Shen, J.; Todd, N.W.; Zhang, H.; Yu, L.; Lingxiao, X.; Mei, Y.; Guarnera, M.; Liao, J.; Chou, A.; Lu, C.L.; et al. Plasma microRNAs as potential biomarkers for non-small-cell lung cancer. *Laboratory investigation; a journal of technical methods and pathology* **2011**, *91*, 579-587, doi:10.1038/labinvest.2010.194.
52. Szczyrek, M.; Kuznar-Kaminska, B.; Grenda, A.; Krawczyk, P.; Sawicki, M.; Glogowski, M.; Balicka, G.; Rolska-Kopinska, A.; Nicos, M.; Jakimiec, M.; et al. Diagnostic value of plasma expression of microRNAs complementary to Drosha and Dicer in lung cancer patients. *European review for medical and pharmacological sciences* **2019**, *23*, 3857-3866, doi:10.26355/eurrev_201905_17813.
53. Niu, Y.; Su, M.; Wu, Y.; Fu, L.; Kang, K.; Li, Q.; Li, L.; Hui, G.; Li, F.; Gou, D. Circulating Plasma miRNAs as Potential Biomarkers of Non-Small Cell Lung Cancer Obtained by High-Throughput Real-Time PCR Profiling. *Cancer epidemiology, biomarkers & prevention : a publication of the American Association for Cancer Research, cosponsored by the American Society of Preventive Oncology* **2019**, *28*, 327-336, doi:10.1158/1055-9965.EPI-18-0723.
54. Arab, A.; Karimipoor, M.; Irani, S.; Kiani, A.; Zeinali, S.; Tafsiri, E.; Sheikhy, K. Potential circulating miRNA signature for early detection of NSCLC. *Cancer genetics* **2017**, *216-217*, 150-158, doi:10.1016/j.cancergen.2017.07.006.
55. Abdollahi, A.; Rahmati, S.; Ghaderi, B.; Sigari, N.; Nikkhoo, B.; Sharifi, K.; Abdi, M. A combined panel of circulating microRNA as a diagnostic tool for detection of the non-small cell lung cancer. *QJM : monthly journal of the Association of Physicians* **2019**, *112*, 779-785, doi:10.1093/qjmed/hcz158.
