# Supplementary material for: A Physically Active Status Affects the Circulating Profile of Cancer-Associated miRNAs
Source: Diagnostics (Basel). 2021 Apr 30;11(5):820. doi: 10.3390/diagnostics11050820 (PMC8147229; doi:10.3390/diagnostics11050820)
Supplement: Supplementary file 1 [file diagnostics-11-00820-s001.zip › Supplementary Table S3.pdf]

diagnostics

Article

A Physically Active Status Affects the Circulating Profile of Cancer-Associated miRNAs

Martina Faraldi [1], Laura Gerosa [2,*], Marta Gomarasca [1], Veronica Sansoni [1], Silvia Perego [1], Ewa Ziemann [3], Giuseppe Banfi [1,4] and Giovanni Lombardi [1,3]

1 IRCCS Istituto Ortopedico Galeazzi, Laboratory of Experimental Biochemistry & Molecular Biology, 20161 Milano, Italy; martina.faraldi@grupposandonato.it (M.F.); marta.gomarasca@grupposandonato.it (M.G.); veronica.sansoni@grupposandonato.it (V.S.); silvia.perego@grupposandonato.it (S.P.); banfi.giuseppe@unisr.it (G.B.); giovanni.lombardi@grupposandonato.it (G.L.)
2 Gruppo San Donato Foundation, 20122 Milano, Italy
3 Department of Athletics, Strength and Conditioning, Poznań University of Physical Education, 61-871 Poznań, Poland; ziemann@awf.poznan.pl
4 Vita-Salute San Raffaele University, 20132 Milano, Italy
* Correspondence: laura.gerosa@grupposandonato.it; Tel.: +39-02166214068

Citation: Faraldi, M.; Gerosa, L.; Gomarasca, M.; Sansoni, V.; Perego, S.; Ziemann, E.; Banfi, G.; Lombardi, G. A Physically Active Status Affects the Circulating Profile of Cancer-Associated miRNAs. *Diagnostics* **2021**, *11*, 820. https://doi.org/10.3390/diagnostics11050820

Academic Editor: Anastasia Malek

Received: 24 March 2021
Accepted: 29 April 2021
Published: 30 April 2021

Publisher's Note: MDPI stays neutral with regard to jurisdictional claims in published maps and institutional affiliations.

Copyright: © 2021 by the authors. Licensee MDPI, Basel, Switzerland. This article is an open access article distributed under the terms and conditions of the Creative Commons Attribution (CC BY) license (http://creativecommons.org/licenses/by/4.0/).

Supplementary Table S3. List of the miRNAs and miRNA signatures associated with diagnosis in Prostate Cancer from literature. Only miRNAs with significant clinically relevance (AUC≥0.7), and signature with at least one modulated miRNA from our exprerimental setting were considered.

Cancer	Sample	miRNAs	miRNA signatures	Regulation	AUC	Reference
PC	Serum	miR-106a<br>miR-223<br>miR-24<br>miR-874<br>miR-93		miR-106a ↑<br>miR-223↓<br>miR-24 ↓<br>miR-874↑<br>miR-93↑	miR-106a: 0.928<br>miR-223: 0.876<br>miR-24: 0.778<br>miR-874: 0.845<br>miR-93: 0.907	[1]
PC	Serum	let-7b<br>miR-141<br>miR-205<br>miR-484		let-7b↓<br>miR-141↑<br>miR-205↓<br>miR-484↓	let-7b: 0.848<br>miR-141: 0.929<br>miR-205: 0.914<br>miR-484: 0.786	[2]
PC	Serum	miR-106b<br>miR-141-3p<br>miR-21<br>miR-375		miR-106b ↑<br>miR-141-3p ↑<br>miR-21 ↑<br>miR-375 ↑	miR-106b: 0.750<br>miR-141-3p: 0.831<br>miR-21: 0.856<br>miR-375: 0.906	[3]
PC	Serum	miR-375		miR-375↑	miR-375: 0.757	[4]
PC	Plasma	miR-145-5p		miR-145-5p ↓	miR-145-5p: 0.900	[5]
PC	Plasma	miR-152-3p<br>miR-326		miR-152-3p ↑<br>miR-326 ↑	miR-326: 0.910<br>miR-152-3p: 0.820	[6]
PC	Plasma	miR-141		miR-141↑	miR-141: 0.800	[7]
PC	Plasma	miR-375		miR-375↓	miR-375: 0.711	[8]
PC	Plasma	miR-21<br>miR-221	miR-21<br>miR-221	miR-21 ↑<br>miR-221 ↑	miR-21: 0.880<br>miR-221: 0.830<br>signature: 0.897	[9]
PC	Plasma	let-7c<br>let-7e<br>miR-30c		let-7c ↓<br>let-7e ↓<br>miR-30c ↓	let-7c: 0.775<br>let-7e: 0.804<br>miR-30c: 0.818	[10]

PC: prostate cancer; AUC: area under the ROC curve.

References

1. Moltzahn, F.; Olshen, A.B.; Baehner, L.; Peek, A.; Fong, L.; Stoppler, H.; Simko, J.; Hilton, J.F.; Carroll, P.; Blelloch, R. Microfluidic-based multiplex qRT-PCR identifies diagnostic and prognostic microRNA signatures in the sera of prostate cancer patients. *Cancer research* **2011**, *71*, 550-560, doi:10.1158/0008-5472.CAN-10-1229.
2. Guo, X.; Han, T.; Hu, P.; Guo, X.; Zhu, C.; Wang, Y.; Chang, S. Five microRNAs in serum as potential biomarkers for prostate cancer risk assessment and therapeutic intervention. *International urology and nephrology* **2018**, *50*, 2193-2200, doi:10.1007/s11255-018-2009-4.
3. Porzycki, P.; Ciszkowicz, E.; Semik, M.; Tyrka, M. Combination of three miRNA (miR-141, miR-21, and miR-375) as potential diagnostic tool for prostate cancer recognition. *International urology and nephrology* **2018**, *50*, 1619-1626, doi:10.1007/s11255-018-1938-2.
4. Wach, S.; Al-Janabi, O.; Weigelt, K.; Fischer, K.; Greither, T.; Marcou, M.; Theil, G.; Nolte, E.; Holzhausen, H.J.; Stohr, R.; et al. The combined serum levels of miR-375 and urokinase plasminogen activator receptor are suggested as diagnostic and prognostic biomarkers in prostate cancer. *International journal of cancer* **2015**, *137*, 1406-1416, doi:10.1002/ijc.29505.
5. Chen, Z.; Zhen, M.; Zhou, J. LncRNA BRE-AS1 interacts with miR-145-5p to regulate cancer cell proliferation and apoptosis in prostate carcinoma and has early diagnostic values. *Bioscience reports* **2019**, *39*, doi:10.1042/BSR20182097.
6. Matin, F.; Jeet, V.; Moya, L.; Selth, L.A.; Chambers, S.; Australian Prostate Cancer, B.; Clements, J.A.; Batra, J. A Plasma Biomarker Panel of Four MicroRNAs for the Diagnosis of Prostate Cancer. *Scientific reports* **2018**, *8*, 6653, doi:10.1038/s41598-018-24424-w.
7. Osipov, I.D.; Zaporozhchenko, I.A.; Bondar, A.A.; Zaripov, M.M.; Voytsitskiy, V.E.; Vlassov, V.V.; Laktionov, P.P.; Morozkin, E.S. Cell-Free miRNA-141 and miRNA-205 as Prostate Cancer Biomarkers. *Advances in experimental medicine and biology* **2016**, *924*, 9-12, doi:10.1007/978-3-319-42044-8_2.
8. Kachakova, D.; Mitkova, A.; Popov, E.; Popov, I.; Vlahova, A.; Dikov, T.; Christova, S.; Mitev, V.; Slavov, C.; Kaneva, R. Combinations of serum prostate-specific antigen and plasma expression levels of let-7c, miR-30c, miR-141, and miR-375 as potential better diagnostic biomarkers for prostate cancer. *DNA and cell biology* **2015**, *34*, 189-200, doi:10.1089/dna.2014.2663.
9. Yaman Agaoglu, F.; Kovancilar, M.; Dizdar, Y.; Darendeliler, E.; Holdenrieder, S.; Dalay, N.; Gezer, U. Investigation of miR-21, miR-141, and miR-221 in blood circulation of patients with prostate cancer. *Tumour biology : the journal of the International Society for Oncodevelopmental Biology and Medicine* **2011**, *32*, 583-588, doi:10.1007/s13277-011-0154-9.
10. Chen, Z.H.; Zhang, G.L.; Li, H.R.; Luo, J.D.; Li, Z.X.; Chen, G.M.; Yang, J. A panel of five circulating microRNAs as potential biomarkers for prostate cancer. *The Prostate* **2012**, *72*, 1443-1452, doi:10.1002/pros.22495.
