# Supplementary material for: A Physically Active Status Affects the Circulating Profile of Cancer-Associated miRNAs
Source: Diagnostics (Basel). 2021 Apr 30;11(5):820. doi: 10.3390/diagnostics11050820 (PMC8147229; doi:10.3390/diagnostics11050820)
Supplement: Supplementary file 1 [file diagnostics-11-00820-s001.zip › Supplementary Table S4.pdf]

diagnostics

MDPI

Article

A Physically Active Status Affects the Circulating Profile of Cancer-Associated miRNAs

Martina Faraldi [1], Laura Gerosa [2,*], Marta Gomarasca [1], Veronica Sansoni [1], Silvia Perego [1], Ewa Ziemann [3], Giuseppe Banfi [1,4] and Giovanni Lombardi [1,3]

1 IRCCS Istituto Ortopedico Galeazzi, Laboratory of Experimental Biochemistry & Molecular Biology, 20161 Milano, Italy; martina.faraldi@grupposandonato.it (M.F.); marta.gomarasca@grupposandonato.it (M.G.); veronica.sansoni@grupposandonato.it (V.S.); silvia.perego@grupposandonato.it (S.P.); banfi.giuseppe@unisr.it (G.B.); giovanni.lombardi@grupposandonato.it (G.L.)

2 Gruppo San Donato Foundation, 20122 Milano, Italy

3 Department of Athletics, Strength and Conditioning, Poznań University of Physical Education, 61-871 Poznań, Poland; ziemann@awf.poznan.pl

4 Vita-Salute San Raffaele University, 20132 Milano, Italy

* Correspondence: laura.gerosa@grupposandonato.it; Tel.: +39-02166214068

Citation: Faraldi, M.; Gerosa, L.; Gomarasca, M.; Sansoni, V.; Perego, S.; Ziemann, E.; Banfi, G.; Lombardi, G. A Physically Active Status Affects the Circulating Profile of Cancer-Associated miRNAs. *Diagnostics* **2021**, *11*, 820. https://doi.org/10.3390/diagnostics11050820

Academic Editor: Anastasia Malek

Received: 24 March 2021
Accepted: 29 April 2021
Published: 30 April 2021

Publisher's Note: MDPI stays neutral with regard to jurisdictional claims in published maps and institutional affiliations.

Copyright: © 2021 by the authors. Licensee MDPI, Basel, Switzerland. This article is an open access article distributed under the terms and conditions of the Creative Commons Attribution (CC BY) license (http://creativecommons.org/licenses/by/4.0/).

Supplementary Table S4. List of the miRNAs and miRNA signatures associated with diagnosis in Colon Cancer from literature. Only miRNAs with significant clinically relevance (AUC≥0.7), and signature with at least one modulated miRNA from our exprerimental setting were considered.

Cancer	Sample	miRNAs	miRNA signatures	Regulation	AUC	Reference
CRC	Serum/ Serum exosomes	miR-21<br>miR-92a		miR-21 ↑ (serum)<br>miR-21 ↑ (serum exosomes)<br>miR-92a ↑ (serum)	serum miR-21: 0.755<br>serum exosome miR-21: 0.770<br>serum miR-92a: 0.747	[1]
CRC	Serum extracellular vesicles	miR-19b<br>miR-21<br>miR-222<br>miR-92a		miR-19b ↑<br>miR-21 ↑<br>miR-222 ↑<br>miR-92a ↑	miR-19b: 0.886<br>miR-21: 0.981<br>miR-222: 0.896<br>miR-92a: 0.951	[2]
CRC	Serum exosomes	let-7a<br>miR-143<br>miR-145<br>miR-150<br>miR-19a<br>miR-20a		let-7a ↑<br>miR-143 ↓<br>miR-145 ↓<br>miR-150 ↑<br>miR-19a ↑<br>miR-20a ↑	let-7a: 0.710<br>miR-143: 0.760<br>miR-145: 0.780<br>miR-150: 0.750<br>miR-19a: 0.870<br>miR-20a: 0.830	[3]
CRC	Serum exosomes	miR-150-5p		miR-150-5p ↓	miR-150-5p: 0.870	[4]
CRC	Serum exosomes	miR-150<br>miR-21<br>miR-223<br>miR-23a		miR-150 ↑<br>miR-21 ↑<br>miR-223 ↑<br>miR-23a ↑	miR-150: 0.758<br>miR-21: 0.798<br>miR-223: 0.716<br>miR-23a: 0.953	[5]
CRC	Serum exosomes	miR-23a<br>miR-301a		miR-23a ↑<br>miR-301a ↑	miR-23a: 0.890<br>miR-301a: 0.840	[6]
CRC	Serum	miR-21<br>miR-210		miR-21 ↑<br>miR-210 ↑	miR-21: 0.973<br>miR-210: 0.934	[7]
CRC	Serum	miR-483-5p		miR-483-5p ↑	miR-483-5p: 0.712	[8]
CRC	Serum	miR-17<br>miR-19a<br>miR-20a<br>miR-223		miR-17 ↑<br>miR-19a ↑<br>miR-201 ↑<br>miR-223 ↑	miR-17: 0.813<br>miR-19a: 0.825<br>miR-20a: 0.788<br>miR-223: 0.838	[9]
CRC	Serum	miR-210		miR-210 ↑	miR-210: 0.821	[10]
CRC	Serum	miR-21-5p	miR-19a-3p<br>miR-21-5p<br>miR-425-5p	miR-19a-3p ↑<br>miR-21-5p ↑<br>miR-425-5p ↑	miR-21-5p: 0.773<br>signature: 0.768	[11]
CRC	Serum	miR-21		miR-21 ↑	miR-21: 0.940	[12]
CRC	Serum	miR-21		miR-21 ↑	miR-21: 0.783	[13]
CRC	Serum	miR-21<br>miR-221<br>miR-92a	miR-155<br>miR-200c<br>miR-21<br>miR-210<br>miR-221<br>miR-29a<br>miR-92a	miR-155 ↑<br>miR-200c ↑<br>miR-21 ↑<br>miR-210 ↑<br>miR-221 ↑<br>miR-29a ↑<br>miR-92a ↑	miR-21: 0.913<br>miR-221: 0.882<br>miR-92a: 0.809<br>signature miR-21, miR-221, miR-92a: 0.891<br>signature miR-155, miR-200c, miR-21, miR-210, miR-29a: 0.890	[14]
CRC	Serum	miR-21-5p		miR-21-5p ↑	miR-21-5p: 0.928	[15]
CRC	Serum	miR-497		miR-497 ↓	miR-497: 0.883	[16]
CRC	Serum	miR-142-3p		miR-142-3p ↓	miR-142-3p: 0.740	[17]
CRC	Serum	miR-143-3p<br>miR-424-5p		miR-143-3p ↓<br>miR-424-5p ↑	miR-143-3p: 0.724<br>miR-424-5p: 0.703	[18]
CRC	Serum	miR-30a-5p		miR-30a-5p ↓	miR-30a-5p: 0.858	[19]
CRC	Serum	miR-199a		miR-199a ↓	miR-199a: 0.864	[20]
CRC	Serum	miR-101		miR-101 ↓	miR-101: 0.730	[21]
CRC	Serum	miR-103		miR-103 ↑	miR-103: 0.857	[22]
CRC	Serum	miR-200c		miR-200c ↑	miR-200c: 0.838	[23]
CRC	Serum	miR-375<br>miR-92a		miR-375 ↓<br>miR-92a ↑	miR-375: 0.781<br>miR-92a: 0.844	[24]
CRC	Serum	miR-194<br>miR-29b		miR-194 ↓<br>miR-29b ↓	miR-194: 0.850<br>miR-29b: 0.870	[25]
CRC	Serum	miR-106a<br>miR-145		miR-106a ↑<br>miR-145 ↓	miR-106a: 0.808<br>miR-145: 0.874	[26]
CRC	Serum	miR-125b<br>miR-21<br>miR-29a	miR-125b<br>miR-21<br>miR-29a	miR-125b ↑<br>miR-21 ↑<br>miR-29a ↑	miR-125b: 0.806<br>miR-21: 0.706<br>miR-29a: 0.741<br>signature: 0.827	[27]
CRC	Serum	miR-145		miR-145 ↓	miR-145: 0.780	[28]
CRC	Serum	miR-155		miR-155 ↑	miR-155: 0.776	[29]
CRC	Serum	miR-19a-3p<br>miR-223-3p<br>miR-92a-3p		miR-19a-3p ↑<br>miR-223-3p ↑<br>miR-92a-3p ↑	miR-19a-3p: 0.849<br>miR-223-3p: 0.890<br>miR-92a-3p: 0.871	[30]

CRC	Serum	miR-21		miR-21 ↑	miR-21: 0.870	[31]
CRC	Serum	miR-21		miR-21 ↑	miR-21: 0.919	[32]
CRC	Serum	miR-21<br>miR-92a	miR-21<br>miR-92a	miR-21 ↑<br>miR-92a ↑	miR-21: 0.802<br>miR-92a: 0.786<br>signature: 0.847	[33]
CRC	Plasma exosomes	miR-92b		miR-92b ↓	miR-92b: 0.793	[34]
CRC	Plasma exosomes	miR-221		miR-221 ↑	miR-221 plasma: 0.781<br>miR-221 exosome: 0.827	[35]
CRC	Plasma exosomes	miR-130a<br>miR-27a	miR-130a<br>miR-27a	miR-130a ↑<br>miR-27a ↑	miR-130a: 0.787<br>miR-27a: 0.82<br>signature: 0.898	[36]
CRC	Plasma	miR-143-3p<br>miR-144-3p<br>miR-148-3p<br>miR-27a-3p<br>miR-424-5p<br>miR-425-5p		miR-143-3p ↓<br>miR-144-3p ↓<br>miR-148-3p ↓<br>miR-27a-3p ↓<br>miR-424-5p ↓<br>miR-425-5p ↓	miR-143-3p: 0.777<br>miR-144-3p: 0.887<br>miR-148-3p:0.871<br>miR-27a-3p: 0.881<br>miR-424-5p: 0.919<br>miR-425-5p: 0.910	[37]
CRC	Plasma	miR-29a<br>miR-92a	miR-29a<br>miR-92a	miR-29a ↑<br>miR-92a ↑	miR-29a: 0.844<br>miR-92a: 0.838<br>signature 0.883	[38]
CRC	Plasma	miR-103a-3p<br>miR-127-3p<br>miR-151a-5p<br>miR-17-5p<br>miR-181a-5p<br>miR-18a-5p<br>miR-18b-5p		miR-103a-3p ↑<br>miR-127-3p ↑<br>miR-151a-5p ↑<br>miR-17-5p ↑<br>miR-181a-5p ↑<br>miR-18a-5p ↑<br>miR-18b-5p ↑	miR-103a-3p: 0.759<br>miR-127-3p: 0.729<br>miR-151a-5p: 0.737<br>miR-17-5p: 0.742<br>miR-181a-5p: 0.736<br>miR-18a-5p: 0.777<br>miR-18b-5p: 0.781	[39]
CRC	Plasma		miR-19a<br>miR-19b<br>miR-15b<br>miR-29a<br>miR-335<br>miR-18a	n.d.	signature: 0.949	[40]
CRC	Plasma	miR-21<br>miR-24		miR-21 ↑<br>miR-24 ↓	miR-21: 0.897<br>miR-24: 0.913	[41]
CRC	Plasma	miR-21	miR-18a<br>miR-21<br>miR-22<br>miR-25	miR-18a↑<br>miR-21 ↑<br>miR-25↑<br>miR-22 ↓	miR-21: 0.840<br>signature: 0.930	[42]
CRC	Plasma	miR-18a<br>miR-191<br>miR-221<br>miR-223<br>miR-24<br>miR-497<br>miR-92a	miR-18a<br>miR-191<br>miR-221<br>miR-223<br>miR-24<br>miR-92a	miR-18a ↑<br>miR-191 ↑<br>miR-221 ↑<br>miR-223 ↑<br>miR-24 ↑<br>miR-497 ↑<br>miR-92a ↑	miR-18a: 0.715<br>miR-191: 0.714<br>miR-221: 0.743<br>miR-223: 0.707<br>miR-24: 0.753<br>miR-497: 0.576<br>miR-92a: 0.751<br>signature miR-18a, miR-191, miR-221, miR-223, miR-24, miR-92a: 0.790<br>signature miR-223, miR-92a: 0.780	[43]
CRC	Plasma	miR-29b		miR-29b ↓	miR-29b: 0.743	[44]
CRC	Plasma	miR-24<br>miR-320a<br>miR-423-5p	miR-24<br>miR-320a<br>miR-423-5p	miR-24 ↓<br>miR-320a ↓<br>miR-423-5p ↓	miR-24: 0.839<br>miR-320a: 0.886<br>miR-423-5p: 0.833<br>signature: 0.899	[45]
CRC	Plasma	miR-375		miR-375 ↓	miR-375: 0.7489	[46]
CRC	Plasma	miR-378		miR-378 ↑	miR-378: 0.796	[47]
CRC	Plasma	miR-25<br>miR-331-3p<br>miR-93	miR-409-3p<br>miR-7<br>miR-93	miR-409-3p ↑<br>miR-7 ↓<br>miR-93 ↓	miR-25: 0.773<br>miR-331-3p: 0.723<br>miR-93: 0.828<br>signature: 0.897	[48]
CRC	Plasma	miR-18a<br>miR-19a<br>miR-19b<br>miR-15b<br>miR-29a<br>miR-335	miR-19a<br>miR-19b<br>miR-15b	miR-18a ↑<br>miR-19a ↑<br>miR-19b ↑<br>miR-15b ↑<br>miR-29a ↑<br>miR-335 ↑	miR-18a: ranging from 0.800 to 0.700<br>miR-19a: ranging from 0.800 to 0.700<br>miR-19b: ranging from 0.800 to 0.700<br>miR-15b: ranging from 0.800 to 0.700<br>miR-29a: ranging from 0.800 to 0.700<br>miR-335: ranging from 0.80 to 0.70<br>signature miR-19a, miR-19b: 0.8194<br>signature miR-19a, miR-19b, miR-15b: 0.8356	[49]
CRC	Plasma	miR-100<br>miR-125b<br>miR-17-3p		miR-100 ↑<br>miR-125b ↑<br>miR-17-3p ↓	miR-100: 0.800<br>miR-125b: 0.890<br>miR-17-3p: 0.710	[50]

CRC	Blood	miR-29a<br>miR-92a<br>miR-23a		miR-29a ↑<br>miR-92a ↑<br>miR-23a ↑	miR-29a: 0.800<br>miR-92a: 0.790<br>miR-23a: 0.787	[51]
CC	Serum	miR-142-5p<br>miR-23a-3p	miR-142-5p<br>miR-23a-3p<br>miR-27a-3p<br>miR-376c-3p	miR-142-5p ↑<br>miR-23a-3p ↑<br>miR-27a-3p ↑<br>miR-376c-3p ↑	miR-142-5p: 0.815<br>miR-23a-3: 0.8908<br>signature: 0.922	[52]
CC	Serum	miR-17<br>miR-21<br>miR-92	miR-17<br>miR-21<br>miR-29a<br>miR-92	miR-17 ↑<br>miR-21 ↑<br>miR-29a ↑<br>miR-92 ↑	miR-17: 0.844<br>miR-21: 0.948<br>miR-92: 0.935<br>signature: 0.881	[53]
CC	Serum	miR-141		miR-141 ↑	miR-141: 0.949	[54]

CRC: colorectal cancer; CC: colon cancer; AUC: area under the ROC curve.

References

1. Uratani, R.; Toiyama, Y.; Kitajima, T.; Kawamura, M.; Hiro, J.; Kobayashi, M.; Tanaka, K.; Inoue, Y.; Mohri, Y.; Mori, T.; et al. Diagnostic Potential of Cell-Free and Exosomal MicroRNAs in the Identification of Patients with High-Risk Colorectal Adenomas. *PloS one* **2016**, *11*, e0160722, doi:10.1371/journal.pone.0160722.
2. de Miguel Perez, D.; Rodriguez Martinez, A.; Ortigosa Palomo, A.; Delgado Urena, M.; Garcia Puche, J.L.; Robles Remacho, A.; Exposito Hernandez, J.; Lorente Acosta, J.A.; Ortega Sanchez, F.G.; Serrano, M.J. Extracellular vesicle-miRNAs as liquid biopsy biomarkers for disease identification and prognosis in metastatic colorectal cancer patients. *Scientific reports* **2020**, *10*, 3974, doi:10.1038/s41598-020-60212-1.
3. Maminezhad, H.; Ghanadian, S.; Pakravan, K.; Razmara, E.; Rouhollah, F.; Mossahebi-Mohammadi, M.; Babashah, S. A panel of six-circulating miRNA signature in serum and its potential diagnostic value in colorectal cancer. *Life sciences* **2020**, *258*, 118226, doi:10.1016/j.lfs.2020.118226.
4. Zou, S.L.; Chen, Y.L.; Ge, Z.Z.; Qu, Y.Y.; Cao, Y.; Kang, Z.X. Downregulation of serum exosomal miR-150-5p is associated with poor prognosis in patients with colorectal cancer. *Cancer biomarkers : section A of Disease markers* **2019**, *26*, 69-77, doi:10.3233/CBM-190156.
5. Ogata-Kawata, H.; Izumiya, M.; Kurioka, D.; Honma, Y.; Yamada, Y.; Furuta, K.; Gunji, T.; Ohta, H.; Okamoto, H.; Sonoda, H.; et al. Circulating exosomal microRNAs as biomarkers of colon cancer. *PloS one* **2014**, *9*, e92921, doi:10.1371/journal.pone.0092921.
6. Karimi, N.; Ali Hosseinpour Feizi, M.; Safaralizadeh, R.; Hashemzadeh, S.; Baradaran, B.; Shokouhi, B.; Teimourian, S. Serum overexpression of miR-301a and miR-23a in patients with colorectal cancer. *Journal of the Chinese Medical Association : JCMA* **2019**, *82*, 215-220, doi:10.1097/JCMA.0000000000000031.
7. Sabry, D.; El-Deek, S.E.M.; Maher, M.; El-Baz, M.A.H.; El-Bader, H.M.; Amer, E.; Hassan, E.A.; Fathy, W.; El-Deek, H.E.M. Role of miRNA-210, miRNA-21 and miRNA-126 as diagnostic biomarkers in colorectal carcinoma: impact of HIF-1alpha-VEGF signaling pathway. *Molecular and cellular biochemistry* **2019**, *454*, 177-189, doi:10.1007/s11010-018-3462-1.
8. Cui, H.; Liu, Y.; Jiang, J.; Liu, Y.; Yang, Z.; Wu, S.; Cao, W.; Cui, I.H.; Yu, C. IGF2-derived miR-483 mediated oncofunction by suppressing DLC-1 and associated with colorectal cancer. *Oncotarget* **2016**, *7*, 48456-48466, doi:10.18632/oncotarget.10309.
9. Zekri, A.R.; Youssef, A.S.; Lotfy, M.M.; Gabr, R.; Ahmed, O.S.; Nassar, A.; Hussein, N.; Omran, D.; Medhat, E.; Eid, S.; et al. Circulating Serum miRNAs as Diagnostic Markers for Colorectal Cancer. *PloS one* **2016**, *11*, e0154130, doi:10.1371/journal.pone.0154130.
10. Wang, W.; Qu, A.; Liu, W.; Liu, Y.; Zheng, G.; Du, L.; Zhang, X.; Yang, Y.; Wang, C.; Chen, X. Circulating miR-210 as a diagnostic and prognostic biomarker for colorectal cancer. *European journal of cancer care* **2017**, *26*, doi:10.1111/ecc.12448.
11. Zhu, M.; Huang, Z.; Zhu, D.; Zhou, X.; Shan, X.; Qi, L.W.; Wu, L.; Cheng, W.; Zhu, J.; Zhang, L.; et al. A panel of microRNA signature in serum for colorectal cancer diagnosis. *Oncotarget* **2017**, *8*, 17081-17091, doi:10.18632/oncotarget.15059.
12. Ghareib, A.F.; Mohamed, R.H.; Abd El-Fatah, A.R.; Saadawy, S.F. Assessment of Serum MicroRNA-21 Gene Expression for Diagnosis and Prognosis of Colorectal Cancer. *Journal of gastrointestinal cancer* **2020**, *51*, 818-823, doi:10.1007/s12029-019-00306-w.
13. Bastaminejad, S.; Taherikalani, M.; Ghanbari, R.; Akbari, A.; Shabab, N.; Saidijam, M. Investigation of MicroRNA-21 Expression Levels in Serum and Stool as a Potential Non-Invasive Biomarker for Diagnosis of Colorectal Cancer. *Iranian biomedical journal* **2017**, *21*, 106-113, doi:10.18869/acadpub.ibj.21.2.106.
14. Pastor-Navarro, B.; Garcia-Flores, M.; Fernandez-Serra, A.; Blanch-Tormo, S.; Martinez de Juan, F.; Martinez-Lapiedra, C.; Maia de Alcantara, F.; Penalver, J.C.; Cervera-Deval, J.; Rubio-Briones, J.; et al. A Tetra-Panel of Serum Circulating miRNAs for the Diagnosis of the Four Most Prevalent Tumor Types. *International journal of molecular sciences* **2020**, *21*, doi:10.3390/ijms21082783.
15. Jin, X.H.; Lu, S.; Wang, A.F. Expression and clinical significance of miR-4516 and miR-21-5p in serum of patients with colorectal cancer. *BMC cancer* **2020**, *20*, 241, doi:10.1186/s12885-020-06715-6.
16. Zou, G.; Wang, R.; Wang, M. Clinical response and prognostic significance of serum miR-497 expression in colorectal cancer. *Cancer biomarkers : section A of Disease markers* **2019**, *25*, 11-18, doi:10.3233/CBM-181902.
17. Gao, W.; Pang, D.; Yu, S. Serum level of miR-142-3p predicts prognostic outcome for colorectal cancer following curative resection. *The Journal of international medical research* **2019**, *47*, 2116-2125, doi:10.1177/0300060519834815.

18. Sahami-Fard, M.H.; Kheirandish, S.; Sheikhha, M.H. Expression levels of miR-143-3p and -424-5p in colorectal cancer and their clinical significance. *Cancer biomarkers : section A of Disease markers* **2019**, *24*, 291-297, doi:10.3233/CBM-182171.
19. Sun, Y.; Yang, B.; Lin, M.; Yu, H.; Chen, H.; Zhang, Z. Identification of serum miR-30a-5p as a diagnostic and prognostic biomarker in colorectal cancer. *Cancer biomarkers : section A of Disease markers* **2019**, *24*, 299-305, doi:10.3233/CBM-182129.
20. Tan, H.Y.; Zheng, Y.B.; Liu, J. Serum miR-199a as a potential diagnostic biomarker for detection of colorectal cancer. *European review for medical and pharmacological sciences* **2018**, *22*, 8657-8663, doi:10.26355/eurrev_201812_16630.
21. He, D.; Yue, Z.; Li, G.; Chen, L.; Feng, H.; Sun, J. Low Serum Levels of miR-101 Are Associated with Poor Prognosis of Colorectal Cancer Patients After Curative Resection. *Medical science monitor : international medical journal of experimental and clinical research* **2018**, *24*, 7475-7481, doi:10.12659/MSM.909768.
22. Wang, D.S.; Zhong, B.; Zhang, M.S.; Gao, Y. Upregulation of serum miR-103 predicts unfavorable prognosis in patients with colorectal cancer. *European review for medical and pharmacological sciences* **2018**, *22*, 4518-4523, doi:10.26355/eurrev_201807_15506.
23. Tayel, S.I.; Fouda, E.A.M.; Gohar, S.F.; Elshayeb, E.I.; El-Sayed, E.H.; El-Kousy, S.M. Potential role of MicroRNA 200c gene expression in assessment of colorectal cancer. *Archives of biochemistry and biophysics* **2018**, *647*, 41-46, doi:10.1016/j.abb.2018.04.009.
24. Elshafei, A.; Shaker, O.; Abd El-Motaal, O.; Salman, T. The expression profiling of serum miR-92a, miR-375, and miR-760 in colorectal cancer: An Egyptian study. *Tumour biology : the journal of the International Society for Oncodevelopmental Biology and Medicine* **2017**, *39*, 1010428317705765, doi:10.1177/1010428317705765.
25. Basati, G.; Razavi, A.E.; Pakzad, I.; Malayeri, F.A. Circulating levels of the miRNAs, miR-194, and miR-29b, as clinically useful biomarkers for colorectal cancer. *Tumour biology : the journal of the International Society for Oncodevelopmental Biology and Medicine* **2016**, *37*, 1781-1788, doi:10.1007/s13277-015-3967-0.
26. Li, J.; Liu, Y.; Wang, C.; Deng, T.; Liang, H.; Wang, Y.; Huang, D.; Fan, Q.; Wang, X.; Ning, T.; et al. Serum miRNA expression profile as a prognostic biomarker of stage II/III colorectal adenocarcinoma. *Scientific reports* **2015**, *5*, 12921, doi:10.1038/srep12921.
27. Yamada, A.; Horimatsu, T.; Okugawa, Y.; Nishida, N.; Honjo, H.; Ida, H.; Kou, T.; Kusaka, T.; Sasaki, Y.; Yagi, M.; et al. Serum miR-21, miR-29a, and miR-125b Are Promising Biomarkers for the Early Detection of Colorectal Neoplasia. *Clinical cancer research : an official journal of the American Association for Cancer Research* **2015**, *21*, 4234-4242, doi:10.1158/1078-0432.CCR-14-2793.
28. Ramzy, I.; Hasaballah, M.; Marzaban, R.; Shaker, O.; Soliman, Z.A. Evaluation of microRNAs-29a, 92a and 145 in colorectal carcinoma as candidate diagnostic markers: An Egyptian pilot study. *Clinics and research in hepatology and gastroenterology* **2015**, *39*, 508-515, doi:10.1016/j.clinre.2014.12.008.
29. Lv, Z.C.; Fan, Y.S.; Chen, H.B.; Zhao, D.W. Investigation of microRNA-155 as a serum diagnostic and prognostic biomarker for colorectal cancer. *Tumour biology : the journal of the International Society for Oncodevelopmental Biology and Medicine* **2015**, *36*, 1619-1625, doi:10.1007/s13277-014-2760-9.
30. Zheng, G.; Du, L.; Yang, X.; Zhang, X.; Wang, L.; Yang, Y.; Li, J.; Wang, C. Serum microRNA panel as biomarkers for early diagnosis of colorectal adenocarcinoma. *British journal of cancer* **2014**, *111*, 1985-1992, doi:10.1038/bjc.2014.489.
31. Basati, G.; Emami Razavi, A.; Abdi, S.; Mirzaei, A. Elevated level of microRNA-21 in the serum of patients with colorectal cancer. *Medical oncology* **2014**, *31*, 205, doi:10.1007/s12032-014-0205-3.
32. Toiyama, Y.; Takahashi, M.; Hur, K.; Nagasaka, T.; Tanaka, K.; Inoue, Y.; Kusunoki, M.; Boland, C.R.; Goel, A. Serum miR-21 as a diagnostic and prognostic biomarker in colorectal cancer. *Journal of the National Cancer Institute* **2013**, *105*, 849-859, doi:10.1093/jnci/djt101.
33. Liu, G.H.; Zhou, Z.G.; Chen, R.; Wang, M.J.; Zhou, B.; Li, Y.; Sun, X.F. Serum miR-21 and miR-92a as biomarkers in the diagnosis and prognosis of colorectal cancer. *Tumour biology : the journal of the International Society for Oncodevelopmental Biology and Medicine* **2013**, *34*, 2175-2181, doi:10.1007/s13277-013-0753-8.
34. Min, L.; Chen, L.; Liu, S.; Yu, Y.; Guo, Q.; Li, P.; Zhu, S. Loss of Circulating Exosomal miR-92b is a Novel Biomarker of Colorectal Cancer at Early Stage. *International journal of medical sciences* **2019**, *16*, 1231-1237, doi:10.7150/ijms.34540.
35. Liu, L.; Meng, T.; Yang, X.H.; Sayim, P.; Lei, C.; Jin, B.; Ge, L.; Wang, H.J. Prognostic and predictive value of long non-coding RNA GAS5 and mircoRNA-221 in colorectal cancer and their effects on colorectal cancer cell proliferation, migration and invasion. *Cancer biomarkers : section A of Disease markers* **2018**, *22*, 283-299, doi:10.3233/CBM-171011.
36. Liu, X.; Pan, B.; Sun, L.; Chen, X.; Zeng, K.; Hu, X.; Xu, T.; Xu, M.; Wang, S. Circulating Exosomal miR-27a and miR-130a Act as Novel Diagnostic and Prognostic Biomarkers of Colorectal Cancer. *Cancer epidemiology, biomarkers & prevention : a publication of the American Association for Cancer Research, cosponsored by the American Society of Preventive Oncology* **2018**, *27*, 746-754, doi:10.1158/1055-9965.EPI-18-0067.
37. Tan, Y.; Lin, J.J.; Yang, X.; Gou, D.M.; Fu, L.; Li, F.R.; Yu, X.F. A panel of three plasma microRNAs for colorectal cancer diagnosis. *Cancer epidemiology* **2019**, *60*, 67-76, doi:10.1016/j.canep.2019.01.015.
38. Huang, Z.; Huang, D.; Ni, S.; Peng, Z.; Sheng, W.; Du, X. Plasma microRNAs are promising novel biomarkers for early detection of colorectal cancer. *International journal of cancer* **2010**, *127*, 118-126, doi:10.1002/ijc.25007.
39. Zhang, H.; Zhu, M.; Shan, X.; Zhou, X.; Wang, T.; Zhang, J.; Tao, J.; Cheng, W.; Chen, G.; Li, J.; et al. A panel of seven-miRNA signature in plasma as potential biomarker for colorectal cancer diagnosis. *Gene* **2019**, *687*, 246-254, doi:10.1016/j.gene.2018.11.055.
40. Herreros-Villanueva, M.; Duran-Sanchon, S.; Martin, A.C.; Perez-Palacios, R.; Vila-Navarro, E.; Marcuello, M.; Diaz-Centeno, M.; Cubiella, J.; Diez, M.S.; Bujanda, L.; et al. Plasma MicroRNA Signature Validation for Early Detection of Colorectal Cancer. *Clinical and translational gastroenterology* **2019**, *10*, e00003, doi:10.14309/ctg.0000000000000003.

41. Ji, C.; Zhao, Y.; Kou, Y.W.; Shao, H.; Guo, L.; Bao, C.H.; Jiang, B.C.; Chen, X.Y.; Dai, J.W.; Tong, Y.X.; et al. Cathepsin F Knockdown Induces Proliferation and Inhibits Apoptosis in Gastric Cancer Cells. *Oncology research* **2018**, *26*, 83-93, doi:10.3727/096504017X14928634401204.
42. Wikberg, M.L.; Myte, R.; Palmqvist, R.; van Guelpen, B.; Ljuslinder, I. Plasma miRNA can detect colorectal cancer, but how early? *Cancer medicine* **2018**, *7*, 1697-1705, doi:10.1002/cam4.1398.
43. Chang, P.Y.; Chen, C.C.; Chang, Y.S.; Tsai, W.S.; You, J.F.; Lin, G.P.; Chen, T.W.; Chen, J.S.; Chan, E.C. MicroRNA-223 and microRNA-92a in stool and plasma samples act as complementary biomarkers to increase colorectal cancer detection. *Oncotarget* **2016**, *7*, 10663-10675, doi:10.18632/oncotarget.7119.
44. Li, L.; Guo, Y.; Chen, Y.; Wang, J.; Zhen, L.; Guo, X.; Liu, J.; Jing, C. The Diagnostic Efficacy and Biological Effects of microRNA-29b for Colon Cancer. *Technology in cancer research & treatment* **2016**, *15*, 772-779, doi:10.1177/1533034615604797.
45. Fang, Z.; Tang, J.; Bai, Y.; Lin, H.; You, H.; Jin, H.; Lin, L.; You, P.; Li, J.; Dai, Z.; et al. Plasma levels of microRNA-24, microRNA-320a, and microRNA-423-5p are potential biomarkers for colorectal carcinoma. *Journal of experimental & clinical cancer research : CR* **2015**, *34*, 86, doi:10.1186/s13046-015-0198-6.
46. Xu, L.; Li, M.; Wang, M.; Yan, D.; Feng, G.; An, G. The expression of microRNA-375 in plasma and tissue is matched in human colorectal cancer. *BMC cancer* **2014**, *14*, 714, doi:10.1186/1471-2407-14-714.
47. Zanutto, S.; Pizzamiglio, S.; Ghilotti, M.; Bertan, C.; Ravagnani, F.; Perrone, F.; Leo, E.; Pilotti, S.; Verderio, P.; Gariboldi, M.; et al. Circulating miR-378 in plasma: a reliable, haemolysis-independent biomarker for colorectal cancer. *British journal of cancer* **2014**, *110*, 1001-1007, doi:10.1038/bjc.2013.819.
48. Wang, S.; Xiang, J.; Li, Z.; Lu, S.; Hu, J.; Gao, X.; Yu, L.; Wang, L.; Wang, J.; Wu, Y.; et al. A plasma microRNA panel for early detection of colorectal cancer. *International journal of cancer* **2015**, *136*, 152-161, doi:10.1002/ijc.28136.
49. Giraldez, M.D.; Lozano, J.J.; Ramirez, G.; Hijona, E.; Bujanda, L.; Castells, A.; Gironella, M. Circulating microRNAs as biomarkers of colorectal cancer: results from a genome-wide profiling and validation study. *Clinical gastroenterology and hepatology : the official clinical practice journal of the American Gastroenterological Association* **2013**, *11*, 681-688 e683, doi:10.1016/j.cgh.2012.12.009.
50. Adams, S.V.; Newcomb, P.A.; Burnett-Hartman, A.N.; Wurscher, M.A.; Mandelson, M.; Upton, M.P.; Zhu, L.C.; Potter, J.D.; Makar, K.W. Rare circulating microRNAs as biomarkers of colorectal neoplasia. *PloS one* **2014**, *9*, e108668, doi:10.1371/journal.pone.0108668.
51. Yong, F.L.; Law, C.W.; Wang, C.W. Potentiality of a triple microRNA classifier: miR-193a-3p, miR-23a and miR-338-5p for early detection of colorectal cancer. *BMC cancer* **2013**, *13*, 280, doi:10.1186/1471-2407-13-280.
52. Vychytilova-Faltejskova, P.; Radova, L.; Sachlova, M.; Kosarova, Z.; Slaba, K.; Fabian, P.; Grolich, T.; Prochazka, V.; Kala, Z.; Svoboda, M.; et al. Serum-based microRNA signatures in early diagnosis and prognosis prediction of colon cancer. *Carcinogenesis* **2016**, *37*, 941-950, doi:10.1093/carcin/bgw078.
53. Conev, N.V.; Donev, I.S.; Konsoulova-Kirova, A.A.; Chervenkov, T.G.; Kashlov, J.K.; Ivanov, K.D. Serum expression levels of miR-17, miR-21, and miR-92 as potential biomarkers for recurrence after adjuvant chemotherapy in colon cancer patients. *Bioscience trends* **2015**, *9*, 393-401, doi:10.5582/bst.2015.01170.
54. Wang, Y.N.; Chen, Z.H.; Chen, W.C. Novel circulating microRNAs expression profile in colon cancer: a pilot study. *European journal of medical research* **2017**, *22*, 51, doi:10.1186/s40001-017-0294-5.
