# Supplementary material for: A Physically Active Status Affects the Circulating Profile of Cancer-Associated miRNAs
Source: Diagnostics (Basel). 2021 Apr 30;11(5):820. doi: 10.3390/diagnostics11050820 (PMC8147229; doi:10.3390/diagnostics11050820)
Supplement: Supplementary file 1 [file diagnostics-11-00820-s001.zip › Supplementary Table S5.pdf]

diagnostics

Article

A Physically Active Status Affects the Circulating Profile of Cancer-Associated miRNAs

Martina Faraldi [1], Laura Gerosa [2,*], Marta Gomarasca [1], Veronica Sansoni [1], Silvia Perego [1], Ewa Ziemann [3], Giuseppe Banfi [1,4] and Giovanni Lombardi [1,3]

1 IRCCS Istituto Ortopedico Galeazzi, Laboratory of Experimental Biochemistry & Molecular Biology, 20161 Milano, Italy; martina.faraldi@grupposandonato.it (M.F.); marta.gomarasca@grupposandonato.it (M.G.); veronica.sansoni@grupposandonato.it (V.S.); silvia.perego@grupposandonato.it (S.P.); banfi.giuseppe@unisr.it (G.B.); giovanni.lombardi@grupposandonato.it (G.L.)
2 Gruppo San Donato Foundation, 20122 Milano, Italy
3 Department of Athletics, Strength and Conditioning, Poznań University of Physical Education, 61-871 Poznań, Poland; ziemann@awf.poznan.pl
4 Vita-Salute San Raffaele University, 20132 Milano, Italy
* Correspondence: laura.gerosa@grupposandonato.it; Tel.: +39-02166214068

Citation: Faraldi, M.; Gerosa, L.; Gomarasca, M.; Sansoni, V.; Perego, S.; Ziemann, E.; Banfi, G.; Lombardi, G. A Physically Active Status Affects the Circulating Profile of Cancer-Associated miRNAs. *Diagnostics* **2021**, *11*, 820. https://doi.org/10.3390/diagnostics11050820

Academic Editor: Anastasia Malek

Received: 24 March 2021
Accepted: 29 April 2021
Published: 30 April 2021

Publisher's Note: MDPI stays neutral with regard to jurisdictional claims in published maps and institutional affiliations.

Copyright: © 2021 by the authors. Licensee MDPI, Basel, Switzerland. This article is an open access article distributed under the terms and conditions of the Creative Commons Attribution (CC BY) license (http://creativecommons.org/licenses/by/4.0/).

Supplementary Table S5. List of the miRNAs and miRNA signatures associated with diagnosis in Liver Cancer from literature. Only miRNAs with significant clinically relevance (AUC≥0.7), and signature with at least one modulated miRNA from our exprerimental setting were considered.

Cancer	Sample	miRNAs	miRNA signatures	Regulation	AUC	Reference
HC	Serum	miR-193a-5p		miR-193a-5p ↑	miR-193a-5p: 0.790	[1]
HC	Serum	miR-143		miR-143 ↓	miR-143: 0.702	[2]
HC	Serum	miR-130b<br>miR-21		miR-130b ↑<br>miR-21 ↑	miR-130b: 0.725<br>miR-21: 0.795	[3]
HC	Serum	miR-122<br>miR-125b<br>miR-126<br>miR-21<br>miR-222<br>miR-30c		miR-122 ↑<br>miR-125b ↑<br>miR-126 ↑<br>miR-21 ↑<br>miR-222 ↑<br>miR-30c ↑	miR-122: 0.959<br>miR-125b: 0.720<br>miR-126: 0.773<br>miR-21: 0.937<br>miR-222: 0.894<br>miR-30c: 0.891	[4]
HC	Serum	miR-107<br>miR-92a-3p		miR-107 ↑<br>miR-92a-3p ↓	miR-107: 0.730<br>miR-92a-3p: 0.705	[5]
HC	Serum	miR-148a<br>miR-152		miR-148a↓<br>miR-152↓	miR-148a: 0.746<br>miR-152: 0.731	[6]
HC	Serum	miR-141<br>miR-200a		miR-141 ↑<br>miR-200a ↑	miR-141: 0.850<br>miR-200a: 0.820	[7]
HC	Serum	miR-130a<br>miR-146a<br>miR-19a<br>miR-192<br>miR-34a		miR-130a ↑<br>miR-146a ↑<br>miR-19a ↓<br>miR-192 ↑<br>miR-34a ↑	miR-130a: 0.910<br>miR-146a: 0.787<br>miR-19a: 0.710<br>miR-192: 0.878<br>miR-34a: 0.980	[8]
HC	Serum	miR-150		miR-150↓	miR-150: 0.931	[9]
HC	Serum	miR-10b<br>miR-106b<br>miR-181a	miR-10b<br>miR-106b<br>miR-181a	miR-10b ↑<br>miR-106b ↑<br>miR-181a ↓	miR-10b: 0.850<br>miR-106b: 0.890<br>miR-181a: 0.820<br>signature: 0.940	[10]
HC	Serum	miR-199a-3p		miR-199a-3p ↓	miR-199a-3p 0.883	[11]
HC	Serum	miR-143<br>miR-215		miR-143 ↑<br>miR-215 ↑	miR-143: 0.795<br>miR-215: 0.816	[12]
HC	Serum	miR-101		miR-101 ↓	miR-101: 0.788	[13]
HC	Serum	miR-221		miR-221 ↑	miR-221: 0.945	[14]
HC	Serum	miR-22		miR-22 ↓	miR-22: 0.866	[15]
HC	Serum	miR-215		miR-215 ↓	miR-215: 0.930	[16]
HC	Serum	miR-122<br>miR-375<br>miR-423		miR-122 ↑<br>miR-375 ↑<br>miR-423 ↑	miR-122: 0.898<br>miR-375: 0.918<br>miR-423: 0.871	[17]
HC	Serum	miR-574-3p		miR-574-3p ↑	miR-574-3p: 0:837	[18]
HC	Serum	miR-125b<br>miR-145<br>miR-375		miR-125b ↑<br>miR-145 ↑<br>miR-375 ↑	miR-125b: 0.702<br>miR-145: 0.892<br>miR-375: 0.741	[19]
HC	Serum		miR-192-5p<br>miR-27b-3p	miR-192-5p ↑<br>miR-27b-3p ↑	signature: 0.823	[20]
HC	Serum	miR-29b	miR-122<br>miR-29b<br>miR-885-5p	miR-122 ↑<br>miR-29b ↓<br>miR-885-5p ↑	miR-29b: 0.766<br>signature: 0.898	[21]
HC	Serum	miR-26a	miR-101<br>miR-21<br>miR-26a	miR-101 ↓<br>miR-21 ↓<br>miR-26a ↓	miR-26a: 0.754<br>signature: 0.803	[22]
HC	Serum	miR-125b<br>miR-223<br>miR-26a<br>miR-27a	miR-125b<br>miR-223<br>miR-26a<br>miR-27a	miR-125b ↓<br>miR-223 ↓<br>miR-26a ↓<br>miR-27a ↓	miR-125b: 0.835<br>miR-223: 0.736<br>miR-27a: 0.859<br>miR-26: 0.711<br>signature: 0.932	[23]
HC	Serum	miR-18a		miR-18a ↑	miR-18a: 0.881	[24]
HC	Serum	miR-23b	miR-23a<br>miR-23b<br>miR-342-3p<br>miR-375<br>miR-423		miR-23b: 0.967<br>signature: 0.999	[25]
HC	Serum	miR-143		miR-143 ↓	miR-143: 0.831	[26]
HC	Serum	miR-122		miR-122 ↓	miR-122: 0.980	[27]
HC	Serum exosomes	miR-106a<br>miR-122<br>miR-17-5p<br>miR-192		miR-106a ↑<br>miR-122 ↑<br>miR-17-5p ↑<br>miR-192 ↑	miR-106a: 0.704<br>miR-122: 0.746<br>miR-17-5p: 0.85<br>miR-192: 0.752	[28]

HC	Serum exosomes	miR-194<br>miR-29a<br>miR-122<br>miR-148a	miR-194 ↑<br>miR-29a ↑<br>miR-122 ↑<br>miR-148a ↑	miR-194: 0.738<br>miR-29a: 0.703<br>miR-122: 0.990<br>miR-148a: 0.871	[29]
HC	Plasma	miR-100-5p<br>miR-122-5p<br>miR-125b-5p<br>miR-574-3p<br>miR-885-5p	miR-100-5p ↑<br>miR-122-5p ↑<br>miR-125b-5p ↑<br>miR-574-3p ↑<br>miR-885-5p ↑	miR-100-5p 0.740<br>miR-122-5p: 0.790<br>miR-125b-5p: 0.770<br>miR-148a-3p: 0.800<br>miR-574-3p: 0.730<br>miR-885-5p: 0.820	[30]
HC	Plasma	miR-15b	miR-15b ↑	miR-15b: 0.871	[31]
HC	Plasma	miR-21	miR-21 ↑	miR-21: 0.953	[32]
HC	Plasma	miR-106b	miR-106b ↑	miR-106b: 0.879	[33]
HC	Plasma	miR-148a	miR-148a ↓	miR-148a: 0.980	[34]
HC	Plasma	miR-122	miR-122 ↓	miR-122: 0.984	[35]
HC	Plasma	miR-483-5p	miR-483-5p ↑	miR-483-5p: 0.827	[36]

HC: hepatocellular carcinoma; AUC: area under the ROC curve

References

1. Loosen, S.H.; Wirtz, T.H.; Roy, S.; Vucur, M.; Castoldi, M.; Schneider, A.T.; Koppe, C.; Ulmer, T.F.; Roeth, A.A.; Bednarsch, J.; et al. Circulating levels of microRNA193a-5p predict outcome in early stage hepatocellular carcinoma. *PloS one* **2020**, *15*, e0239386, doi:10.1371/journal.pone.0239386.
2. Aly, D.M.; Gohar, N.A.; Abd El-Hady, A.A.; Khairy, M.; Abdullatif, M.M. Serum microRNA let-7a-1/let-7d/let-7f and miRNA 143/145 Gene Expression Profiles as Potential Biomarkers in HCV Induced Hepatocellular Carcinoma. *Asian Pacific journal of cancer prevention : APJCP* **2020**, *21*, 555-562, doi:10.31557/APJCP.2020.21.2.555.
3. Zhang, N.; Hu, Z.; Qiang, Y.; Zhu, X. Circulating miR-130b- and miR-21-based diagnostic markers and therapeutic targets for hepatocellular carcinoma. *Molecular genetics & genomic medicine* **2019**, *7*, e1012, doi:10.1002/mgg3.1012.
4. Ali, H.E.A.; Abdel Hameed, R.; Effat, H.; Ahmed, E.K.; Atef, A.A.; Sharawi, S.K.; Ali, M.; Abd Elmageed, Z.Y.; Abdel Wahab, A.H. Circulating microRNAs panel as a diagnostic tool for discrimination of HCV-associated hepatocellular carcinoma. *Clinics and research in hepatology and gastroenterology* **2017**, *41*, e51-e62, doi:10.1016/j.clinre.2017.06.004.
5. Zhang, Y.; Li, T.; Qiu, Y.; Zhang, T.; Guo, P.; Ma, X.; Wei, Q.; Han, L. Serum microRNA panel for early diagnosis of the onset of hepatocellular carcinoma. *Medicine* **2017**, *96*, e5642, doi:10.1097/MD.0000000000005642.
6. Wang, F.; Ying, H.; He, B.; Pan, Y.; Sun, H.; Wang, S. Circulating miR-148/152 family as potential biomarkers in hepatocellular carcinoma. *Tumour biology : the journal of the International Society for Oncodevelopmental Biology and Medicine* **2016**, *37*, 4945-4953, doi:10.1007/s13277-015-4340-z.
7. Dhayat, S.A.; Husing, A.; Senninger, N.; Schmidt, H.H.; Haier, J.; Wolters, H.; Kabar, I. Circulating microRNA-200 Family as Diagnostic Marker in Hepatocellular Carcinoma. *PloS one* **2015**, *10*, e0140066, doi:10.1371/journal.pone.0140066.
8. Motawi, T.K.; Shaker, O.G.; El-Maraghy, S.A.; Senousy, M.A. Serum MicroRNAs as Potential Biomarkers for Early Diagnosis of Hepatitis C Virus-Related Hepatocellular Carcinoma in Egyptian Patients. *PloS one* **2015**, *10*, e0137706, doi:10.1371/journal.pone.0137706.
9. Yu, F.; Lu, Z.; Chen, B.; Dong, P.; Zheng, J. microRNA-150: a promising novel biomarker for hepatitis B virus-related hepatocellular carcinoma. *Diagnostic pathology* **2015**, *10*, 129, doi:10.1186/s13000-015-0369-y.
10. Jiang, L.; Cheng, Q.; Zhang, B.H.; Zhang, M.Z. Circulating microRNAs as biomarkers in hepatocellular carcinoma screening: a validation set from China. *Medicine* **2015**, *94*, e603, doi:10.1097/MD.0000000000000603.
11. Yin, J.; Hou, P.; Wu, Z.; Wang, T.; Nie, Y. Circulating miR-375 and miR-199a-3p as potential biomarkers for the diagnosis of hepatocellular carcinoma. *Tumour biology : the journal of the International Society for Oncodevelopmental Biology and Medicine* **2015**, *36*, 4501-4507, doi:10.1007/s13277-015-3092-0.
12. Zhang, Z.Q.; Meng, H.; Wang, N.; Liang, L.N.; Liu, L.N.; Lu, S.M.; Luan, Y. Serum microRNA 143 and microRNA 215 as potential biomarkers for the diagnosis of chronic hepatitis and hepatocellular carcinoma. *Diagnostic pathology* **2014**, *9*, 135, doi:10.1186/1746-1596-9-135.
13. Xie, Y.; Yao, Q.; Butt, A.M.; Guo, J.; Tian, Z.; Bao, X.; Li, H.; Meng, Q.; Lu, J. Expression profiling of serum microRNA-101 in HBV-associated chronic hepatitis, liver cirrhosis, and hepatocellular carcinoma. *Cancer biology & therapy* **2014**, *15*, 1248-1255, doi:10.4161/cbt.29688.
14. Li, F.; Wang, F.; Zhu, C.; Wei, Q.; Zhang, T.; Zhou, Y.L. miR-221 suppression through nanoparticle-based miRNA delivery system for hepatocellular carcinoma therapy and its diagnosis as a potential biomarker. *International journal of nanomedicine* **2018**, *13*, 2295-2307, doi:10.2147/IJN.S157805.
15. Zeng, Z.; Dong, J.; Li, Y.; Dong, Z.; Liu, Z.; Huang, J.; Wang, Y.; Zhen, Y.; Lu, Y. The expression level and diagnostic value of microRNA-22 in HC patients. *Artificial cells, nanomedicine, and biotechnology* **2020**, *48*, 683-686, doi:10.1080/21691401.2019.1703723.
16. El Mahdy, H.A.; Abdelhamid, I.A.; Amen, A.I.; Abdelsameea, E.; Hassouna, M.M. MicroRNA-215 as a Diagnostic Marker in Egyptian Patients with Hepatocellular Carcinoma. *Asian Pacific journal of cancer prevention : APJCP* **2019**, *20*, 2723-2731, doi:10.31557/APJCP.2019.20.9.2723.

17. An, Y.; Gao, S.; Zhao, W.C.; Qiu, B.A.; Xia, N.X.; Zhang, P.J.; Fan, Z.P. Novel serum microRNAs panel on the diagnostic and prognostic implications of hepatocellular carcinoma. *World journal of gastroenterology* **2018**, *24*, 2596-2604, doi:10.3748/wjg.v24.i24.2596.
18. Shen, X.; Xue, Y.; Cong, H.; Wang, X.; Ju, S. Dysregulation of serum microRNA-574-3p and its clinical significance in hepatocellular carcinoma. *Annals of clinical biochemistry* **2018**, *55*, 478-484, doi:10.1177/0004563217741908.
19. Elemeery, M.N.; Badr, A.N.; Mohamed, M.A.; Ghareeb, D.A. Validation of a serum microRNA panel as biomarkers for early diagnosis of hepatocellular carcinoma post-hepatitis C infection in Egyptian patients. *World journal of gastroenterology* **2017**, *23*, 3864-3875, doi:10.3748/wjg.v23.i21.3864.
20. Zhu, H.T.; Liu, R.B.; Liang, Y.Y.; Hasan, A.M.E.; Wang, H.Y.; Shao, Q.; Zhang, Z.C.; Wang, J.; He, C.Y.; Wang, F.; et al. Serum microRNA profiles as diagnostic biomarkers for HBV-positive hepatocellular carcinoma. *Liver international : official journal of the International Association for the Study of the Liver* **2017**, *37*, 888-896, doi:10.1111/liv.13356.
21. Zekri, A.N.; Youssef, A.S.; El-Desouky, E.D.; Ahmed, O.S.; Lotfy, M.M.; Nassar, A.A.; Bahnassey, A.A. Serum microRNA panels as potential biomarkers for early detection of hepatocellular carcinoma on top of HCV infection. *Tumour biology : the journal of the International Society for Oncodevelopmental Biology and Medicine* **2016**, *37*, 12273-12286, doi:10.1007/s13277-016-5097-8.
22. Zhuang, C.; Jiang, W.; Huang, D.; Xu, L.; Yang, Q.; Zheng, L.; Wang, X.; Hu, L. Serum miR-21, miR-26a and miR-101 as potential biomarkers of hepatocellular carcinoma. *Clinics and research in hepatology and gastroenterology* **2016**, *40*, 386-396, doi:10.1016/j.clinre.2015.11.002.
23. Zuo, D.; Chen, L.; Liu, X.; Wang, X.; Xi, Q.; Luo, Y.; Zhang, N.; Guo, H. Combination of miR-125b and miR-27a enhances sensitivity and specificity of AFP-based diagnosis of hepatocellular carcinoma. *Tumour biology : the journal of the International Society for Oncodevelopmental Biology and Medicine* **2016**, *37*, 6539-6549, doi:10.1007/s13277-015-4545-1.
24. Li, L.; Guo, Z.; Wang, J.; Mao, Y.; Gao, Q. Serum miR-18a: a potential marker for hepatitis B virus-related hepatocellular carcinoma screening. *Digestive diseases and sciences* **2012**, *57*, 2910-2916, doi:10.1007/s10620-012-2317-y.
25. Li, L.M.; Hu, Z.B.; Zhou, Z.X.; Chen, X.; Liu, F.Y.; Zhang, J.F.; Shen, H.B.; Zhang, C.Y.; Zen, K. Serum microRNA profiles serve as novel biomarkers for HBV infection and diagnosis of HBV-positive hepatocarcinoma. *Cancer research* **2010**, *70*, 9798-9807, doi:10.1158/0008-5472.CAN-10-1001.
26. Zhang, J.; Lin, H.; Wang, X.Y.; Zhang, D.Q.; Chen, J.X.; Zhuang, Y.; Zheng, X.L. Predictive value of microRNA-143 in evaluating the prognosis of patients with hepatocellular carcinoma. *Cancer biomarkers : section A of Disease markers* **2017**, *19*, 257-262, doi:10.3233/CBM-160357.
27. Shehab-Eldeen, S.; Nada, A.; Abou-Elela, D.; El-Naidany, S.; Arafat, E.; Omar, T. Diagnostic Performance of microRNA-122 and microRNA-224 in Hepatitis C Virus-Induced Hepatocellular Carcinoma (HC). *Asian Pacific journal of cancer prevention : APJCP* **2019**, *20*, 2515-2522, doi:10.31557/APJCP.2019.20.8.2515.
28. Xue, X.; Zhao, Y.; Wang, X.; Qin, L.; Hu, R. Development and validation of serum exosomal microRNAs as diagnostic and prognostic biomarkers for hepatocellular carcinoma. *Journal of cellular biochemistry* **2019**, *120*, 135-142, doi:10.1002/jcb.27165.
29. Wang, Y.; Zhang, C.; Zhang, P.; Guo, G.; Jiang, T.; Zhao, X.; Jiang, J.; Huang, X.; Tong, H.; Tian, Y. Serum exosomal microRNAs combined with alpha-fetoprotein as diagnostic markers of hepatocellular carcinoma. *Cancer medicine* **2018**, *7*, 1670-1679, doi:10.1002/cam4.1390.
30. Jin, Y.; Wong, Y.S.; Goh, B.K.P.; Chan, C.Y.; Cheow, P.C.; Chow, P.K.H.; Lim, T.K.H.; Goh, G.B.B.; Krishnamoorthy, T.L.; Kumar, R.; et al. Circulating microRNAs as Potential Diagnostic and Prognostic Biomarkers in Hepatocellular Carcinoma. *Scientific reports* **2019**, *9*, 10464, doi:10.1038/s41598-019-46872-8.
31. Chen, Y.; Chen, J.; Liu, Y.; Li, S.; Huang, P. Plasma miR-15b-5p, miR-338-5p, and miR-764 as Biomarkers for Hepatocellular Carcinoma. *Medical science monitor : international medical journal of experimental and clinical research* **2015**, *21*, 1864-1871, doi:10.12659/MSM.893082.
32. Tomimaru, Y.; Eguchi, H.; Nagano, H.; Wada, H.; Kobayashi, S.; Marubashi, S.; Tanemura, M.; Tomokuni, A.; Takemasa, I.; Umeshita, K.; et al. Circulating microRNA-21 as a novel biomarker for hepatocellular carcinoma. *Journal of hepatology* **2012**, *56*, 167-175, doi:10.1016/j.jhep.2011.04.026.
33. Jiang, L.; Li, X.; Cheng, Q.; Zhang, B.H. Plasma microRNA might as a potential biomarker for hepatocellular carcinoma and chronic liver disease screening. *Tumour biology : the journal of the International Society for Oncodevelopmental Biology and Medicine* **2015**, *36*, 7167-7174, doi:10.1007/s13277-015-3446-7.
34. Han, J.; Li, J.; Qian, Y.; Liu, W.; Liang, J.; Huang, Z.; Wang, S.; Zhao, C. Identification of plasma miR-148a as a noninvasive biomarker for hepatocellular carcinoma. *Clinics and research in hepatology and gastroenterology* **2019**, *43*, 585-593, doi:10.1016/j.clinre.2018.12.008.
35. Quoc, N.B.; Phuong, N.D.N.; Ngan, T.K.; Linh, N.T.M.; Cuong, P.H.; Chau, N.N.B. Expression of Plasma hsa-miR122 in HBV-Related Hepatocellular Carcinoma (HC) in Vietnamese Patients. *MicroRNA* **2018**, *7*, 92-99, doi:10.2174/2211536607666180427165114.
36. Shen, J.; Wang, A.; Wang, Q.; Gurvich, I.; Siegel, A.B.; Remotti, H.; Santella, R.M. Exploration of genome-wide circulating microRNA in hepatocellular carcinoma: MiR-483-5p as a potential biomarker. *Cancer epidemiology, biomarkers & prevention : a publication of the American Association for Cancer Research, cosponsored by the American Society of Preventive Oncology* **2013**, *22*, 2364-2373, doi:10.1158/1055-9965.EPI-13-0237.
