# Supplementary material for: A Physically Active Status Affects the Circulating Profile of Cancer-Associated miRNAs
Source: Diagnostics (Basel). 2021 Apr 30;11(5):820. doi: 10.3390/diagnostics11050820 (PMC8147229; doi:10.3390/diagnostics11050820)
Supplement: Supplementary file 1 [file diagnostics-11-00820-s001.zip › Supplementary Table S6.pdf]

diagnostics

MDPI

Article

A Physically Active Status Affects the Circulating Profile of Cancer-Associated miRNAs

Martina Faraldi [1], Laura Gerosa [2,*], Marta Gomarasca [1], Veronica Sansoni [1], Silvia Perego [1], Ewa Ziemann [3], Giuseppe Banfi [1,4] and Giovanni Lombardi [1,3]

1 IRCCS Istituto Ortopedico Galeazzi, Laboratory of Experimental Biochemistry & Molecular Biology, 20161 Milano, Italy; martina.faraldi@grupposandonato.it (M.F.); marta.gomarasca@grupposandonato.it (M.G.); veronica.sansoni@grupposandonato.it (V.S.); silvia.perego@grupposandonato.it (S.P.); banfi.giuseppe@unisr.it (G.B.); giovanni.lombardi@grupposandonato.it (G.L.)
2 Gruppo San Donato Foundation, 20122 Milano, Italy
3 Department of Athletics, Strength and Conditioning, Poznań University of Physical Education, 61-871 Poznań, Poland; ziemann@awf.poznan.pl
4 Vita-Salute San Raffaele University, 20132 Milano, Italy
* Correspondence: laura.gerosa@grupposandonato.it; Tel.: +39-02166214068

Citation: Faraldi, M.; Gerosa, L.; Gomarasca, M.; Sansoni, V.; Perego, S.; Ziemann, E.; Banfi, G.; Lombardi, G. A Physically Active Status Affects the Circulating Profile of Cancer-Associated miRNAs. *Diagnostics* **2021**, *11*, 820. https://doi.org/10.3390/diagnostics11050820

Academic Editor: Anastasia Malek

Received: 24 March 2021
Accepted: 29 April 2021
Published: 30 April 2021

Publisher's Note: MDPI stays neutral with regard to jurisdictional claims in published maps and institutional affiliations.

Copyright: © 2021 by the authors. Licensee MDPI, Basel, Switzerland. This article is an open access article distributed under the terms and conditions of the Creative Commons Attribution (CC BY) license (http://creativecommons.org/licenses/by/4.0/).

Supplementary Table S6. List of the miRNAs and miRNA signatures associated with diagnosis in Gastro-esophageal Cancer from literature. Only miRNAs with significant clinically relevance (AUC≥0.7), and signature with at least one modulated miRNA from our exprerimental setting were considered.

Cancer	Sample	miRNAs	miRNA signatures	Regulation	AUC	Reference
GC	Serum exosome	miR-423-5p		miR-423-5p ↑	miR-423-5p: 0.763	[1]
GC	Serum exosome	miR-106a-5p<br>miR-19b-3p	miR-106a-5p<br>miR-17-5p<br>miR-19b-3p<br>miR-30a-5p	miR-106a-5p ↑<br>miR-17-5p ns<br>miR-19b ↑<br>miR-30a-5p n.d.	miR-106a-5p: 0.786<br>miR-19b-3p: 0.769<br>signature: 0.814	[2]
GC	Serum	miR-375		miR-375 ↓	miR-375: 0.835	[3]
GC	Serum	miR-101-3p		miR-101-3p ↓	miR-101-3p: 0.875	[4]
GC	Serum	miR-25		miR-25 ↑	miR-25: 0.768	[5]
GC	Serum	miR-21		miR-21 ↑	miR-21: 0.912	[6]
GC	Serum	miR-130a		miR-130a ↑	miR-130a: 0.870	[7]
GC	Serum	miR-100<br>miR-16<br>miR-223		miR-100 ↑<br>miR-16 ↑<br>miR-223 ↑	miR-100: 0.710<br>miR-16: 0.900<br>miR-223: 0.900	[8]
GC	Serum	miR-191		miR-191 ↑	miR-191: 0.849	[9]
GC	Serum	let-7e<br>miR-221<br>miR-376c		let-7e ↑<br>miR-221 ↑<br>miR-376c ↑	let-7e: 0.700<br>miR-221: 0.700<br>miR-376c: 0.710	[10]
GC	Serum	miR-378		miR-378 ↑	miR-378: 0.861	[11]
GC	Serum		miR-1<br>miR-20a<br>miR-27a<br>miR-34<br>miR-423-5p	miR-1 ↑<br>miR-20a ↑<br>miR-27a ↑<br>miR-34 ↑<br>miR-423-5p ↑	signature: 0.831	[12]
GC	Plasma	miR-18a		miR-18a ↑	miR-18a: 0.806	[13]
GC	Plasma	miR-106a<br>miR-106b<br>miR-21<br>miR-93	miR-106a<br>miR-106b<br>miR-21<br>miR-93	miR-106a ↑<br>miR-106b ↑<br>miR-21 ↑<br>miR-93 ↑	miR-106a: 0.731<br>miR-106b: 0.770<br>miR-21: 0.812<br>miR-93: 0.751<br>signature: 0.887	[14]
GC	Plasma	miR-21<br>miR-222		miR-21 ↑<br>miR-222 ↑	miR-21: 0.893<br>miR-222: 0.747	[15]
GC	Plasma	miR-199a-3p		miR-199a-3p ↑	miR-199a-3p: 0.818	[16]
GC	Plasma	miR-27-3p		miR-27-3p ↓	miR-27-3p: 0.724	[17]
GC	Plasma	miR-107<br>miR-194<br>miR-210		miR-107 ↓<br>miR-194 ↓<br>miR-210 ↓	miR-107: 0.947<br>miR-194-5p: 0.862<br>miR-210-3p: 0.820	[18]
GC	Plasma	miR-376c		miR-376c ↑	miR-376c: 0.770	[19]
GC	Plasma	miR-142-3p<br>miR-148a<br>miR-195<br>miR-26a		miR-142-3p ↓<br>miR-148a ↓<br>miR-195 ↓<br>miR-26a ↓	miR-142-3p 0.839<br>miR-148a 0.842<br>miR-195 0.765<br>miR-26a 0.882	[20]
GC	Plasma	miR-23b		miR-23b ↑	miR-23b: 0.800	[21]
GC	Plasma	miR-106a		miR-106a ↑	miR-106a: 0.895	[22]
GC	Plasma	miR-140-5p<br>miR-18a<br>miR-199a-3p<br>miR-629<br>miR-652	miR-629<br>miR-652	miR-140-5p ↑<br>miR-18a ↑<br>miR-199a-3p ↑<br>miR-629 ↑<br>miR-652 ↑	miR-140-5p: 0.850<br>miR-18a: 0.849<br>miR-199a-3p: 0.881<br>miR-629-5p: 0.912<br>miR-652-3p: 0.918<br>signature miR-629, miR-652: 0.963	[23]
GC	Plasma	miR-222		miR-222 ↑	miR-222: 0.850	[24]
GC	Plasma	miR-16<br>miR-25<br>miR-451<br>miR-486-5p<br>miR-92a	miR-16<br>miR-25<br>miR-451<br>miR-486-5p<br>miR-92a	miR-16 ↑<br>miR-25 ↑<br>miR-451 ↑<br>miR-486-5p ↑<br>miR-92a ↑	miR-16: 0.768<br>miR-25: 0.694<br>miR-451: 0.790<br>miR-486-5p: 0.779<br>miR-92a: 0.732<br>signature: 0.812	[25]
GC	Plasma	miR-106b<br>miR-20a<br>miR-221		miR-106b ↑<br>miR-20a ↑<br>miR-221 ↑	miR-106b: 0.773<br>miR-20a: 0.859<br>miR-221: 0.796	[26]
GC	Plasma	miR-199a		miR-199a ↑	miR-199a: 0.837	[27]
GC	Plasma	miR-21<br>miR-223		miR-21 ↑<br>miR-223 ↑	miR-21: 0.743<br>miR-223: 0.909	[28]
GC	Plasma	miR-106b		miR-106b ↑	miR-106b: 0.721	[29]
GC	Plasma	miR-106b<br>miR-25<br>miR-93		miR-106b ↑<br>miR-25 ↑<br>miR-93 ↑	miR-106b: 0.898<br>miR-25: 0.817<br>miR-93: 0.756	[30]

GC	PBMC	miR-17	miR-106a<br>miR-17	miR-106a ↑<br>miR-17 ↑	miR-17: 0.743<br>signature: 0.741	[31]
GC	Blood	miR-200c		miR-200c ↑	miR-200c: 0.715	[32]
EC	Serum	miR-107<br>miR-144<br>miR-152<br>miR-21<br>miR-342<br>miR-93	miR-107<br>miR-144<br>miR-152<br>miR-21<br>miR-342<br>miR-93	miR-107 ↓<br>miR-144 ↑<br>miR-152 ↓<br>miR-21 ↑<br>miR-342 ↓<br>miR-93 ↓	miR-107: 0.554<br>miR-144: 0.677<br>miR-152: 0.536<br>miR-21: 0.692<br>miR-342: 0.817<br>miR-93: 0.677<br>signature: 0.968	[33]
EC	Serum	miR-144		miR-144 ↑	miR-144: 0.731	[34]
EC	Plasma		miR-133a-3p<br>miR-382-5p<br>miR-451a	miR-133a-3p ↓<br>miR-382-5p ↑<br>miR-451a n.d.	signature: 0.846	[35]
EC<br>ESCC	Plasma	miR-19a		miR-19a ↑	miR-19a EC: 0.767<br>miR-19a ESCC: 0.712	[36]
EC	Blood	miR-18a<br>miR-21		miR-18a ↑<br>miR-21 ↑	miR-18a: 0.948<br>miR-21: 0.913	[37]
ESCC	Serum	miR-16-5p		miR-16-5p ↑	miR-16-5p: 0.759	[38]
ESCC	Serum	miR-483-5p		miR-483-5p ↑	miR-483-5p: 0.927	[39]
ESCC	Serum	miR-20b-5p<br>miR-223-3p		miR-20b-5p ↑<br>miR-223-3p ↑	miR-20b-5p: 0.731<br>miR-223-3p: 0.736	[40]
ESCC	Serum	miR-15a		miR-15a ↓	miR-15a: 0.951	[41]
ESCC	Serum	miR-146a		miR-146a ↓	miR-146a: 0.891	[42]
ESCC	Serum	miR-7		miR-7 ↓	miR-7: 0.841	[43]
ESCC	Serum	miR-10b<br>miR-205<br>miR-29c		miR-10b ↑<br>miR-205 ↓<br>miR-29c ↓	miR-10b: 0.850<br>miR-205: 0.880<br>miR-29c: 0.890	[44]
ESCC	Serum	miR-100<br>miR-194<br>miR-223<br>miR-25<br>miR-483-5p		miR-100 ↑<br>miR-194 ↑<br>miR-223 ↑<br>miR-25 ↑<br>miR-483-5p ↑	miR-100: 0.747<br>miR-194: 0.809<br>miR-223: 0.772<br>miR-25: 0.777<br>miR-483-5p: 0.739	[45]
ESCC	Serum	miR-223<br>miR-375	miR-223<br>miR-375	miR-223 ↑<br>miR-375 ↓	miR-223: 0.734<br>miR-375: 0.720<br>signature: 0.745	[46]
ESCC	Serum	miR-100<br>miR-127-3p<br>miR-133a<br>miR-148b<br>miR-22		miR-100 ↑<br>miR-127-3p ↑<br>miR-133a ↑<br>miR-148b ↑<br>miR-22 ↑	miR-100: 0.817<br>miR-127-3p: 0.899<br>miR-133a: 0.830<br>miR-148b: 0.855<br>miR-22: 0.949	[47]
ESCC	Plasma	miR-223<br>miR-375		miR-223 ↑<br>miR-375 ↓	miR-223: 0.911<br>miR-375: 0.807	[48]
ESCC	Plasma	miR-21<br>miR-93		miR-21 ↑<br>miR-93 ↑	miR-21 T stage: 0.819<br>miR-21 N stage: 0.758<br>miR-21 M stage: 0.824<br>miR-21 pathological differentiation: 0.725<br>miR-93 T stage: 0.827<br>miR-93 N stage: 0.815<br>miR-93 M stage: 0.814	[49]
ESCC	Plasma	let-7<br>miR-20a		let-7 ↓<br>miR-20a ↑	let-7: 0.829<br>miR-20a: 0.767	[50]

GC: gastric cancer; EC: esophageal cancer; ESCC: esophageal squamous cell carcinoma; PBMC: peripheral blood mononuclear cells; AUC: area under ROC curve.

References

1. Yang, H.; Fu, H.; Wang, B.; Zhang, X.; Mao, J.; Li, X.; Wang, M.; Sun, Z.; Qian, H.; Xu, W. Exosomal miR-423-5p targets SUFU to promote cancer growth and metastasis and serves as a novel marker for gastric cancer. *Molecular carcinogenesis* **2018**, *57*, 1223-1236, doi:10.1002/mc.22838.
2. Wang, N.; Wang, L.; Yang, Y.; Gong, L.; Xiao, B.; Liu, X. A serum exosomal microRNA panel as a potential biomarker test for gastric cancer. *Biochemical and biophysical research communications* **2017**, *493*, 1322-1328, doi:10.1016/j.bbrc.2017.10.003.
3. Zhang, W.H.; Gui, J.H.; Wang, C.Z.; Chang, Q.; Xu, S.P.; Cai, C.H.; Li, Y.N.; Tian, Y.P.; Yan, L.; Wu, B. The identification of miR-375 as a potential biomarker in distal gastric adenocarcinoma. *Oncology research* **2012**, *20*, 139-147, doi:10.3727/096504012x13522227232156.
4. Zeng, W.; Zhang, S.; Yang, L.; Wei, W.; Gao, J.; Guo, N.; Wu, F. Serum miR-101-3p combined with pepsinogen contributes to the early diagnosis of gastric cancer. *BMC medical genetics* **2020**, *21*, 28, doi:10.1186/s12881-020-0967-8.

5. Kong, Y.; Ning, L.; Qiu, F.; Yu, Q.; Cao, B. Clinical significance of serum miR-25 as a diagnostic and prognostic biomarker in human gastric cancer. *Cancer biomarkers : section A of Disease markers* **2019**, *24*, 477-483, doi:10.3233/CBM-182213.
6. Wu, J.; Li, G.; Wang, Z.; Yao, Y.; Chen, R.; Pu, X.; Wang, J. Circulating MicroRNA-21 Is a Potential Diagnostic Biomarker in Gastric Cancer. *Disease markers* **2015**, *2015*, 435656, doi:10.1155/2015/435656.
7. Jiang, H.; Yu, W.W.; Wang, L.L.; Peng, Y. miR-130a acts as a potential diagnostic biomarker and promotes gastric cancer migration, invasion and proliferation by targeting RUNX3. *Oncology reports* **2015**, *34*, 1153-1161, doi:10.3892/or.2015.4099.
8. Wang, H.; Wang, L.; Wu, Z.; Sun, R.; Jin, H.; Ma, J.; Liu, L.; Ling, R.; Yi, J.; Wang, L.; et al. Three dysregulated microRNAs in serum as novel biomarkers for gastric cancer screening. *Medical oncology* **2014**, *31*, 298, doi:10.1007/s12032-014-0298-8.
9. Peng, W.Z.; Ma, R.; Wang, F.; Yu, J.; Liu, Z.B. Role of miR-191/425 cluster in tumorigenesis and diagnosis of gastric cancer. *International journal of molecular sciences* **2014**, *15*, 4031-4048, doi:10.3390/ijms15034031.
10. Song, M.Y.; Pan, K.F.; Su, H.J.; Zhang, L.; Ma, J.L.; Li, J.Y.; Yuasa, Y.; Kang, D.; Kim, Y.S.; You, W.C. Identification of serum microRNAs as novel non-invasive biomarkers for early detection of gastric cancer. *PloS one* **2012**, *7*, e33608, doi:10.1371/journal.pone.0033608.
11. Liu, H.; Zhu, L.; Liu, B.; Yang, L.; Meng, X.; Zhang, W.; Ma, Y.; Xiao, H. Genome-wide microRNA profiles identify miR-378 as a serum biomarker for early detection of gastric cancer. *Cancer letters* **2012**, *316*, 196-203, doi:10.1016/j.canlet.2011.10.034.
12. Liu, R.; Zhang, C.; Hu, Z.; Li, G.; Wang, C.; Yang, C.; Huang, D.; Chen, X.; Zhang, H.; Zhuang, R.; et al. A five-microRNA signature identified from genome-wide serum microRNA expression profiling serves as a fingerprint for gastric cancer diagnosis. *European journal of cancer* **2011**, *47*, 784-791, doi:10.1016/j.ejca.2010.10.025.
13. Tsujiura, M.; Komatsu, S.; Ichikawa, D.; Shiozaki, A.; Konishi, H.; Takeshita, H.; Moriumura, R.; Nagata, H.; Kawaguchi, T.; Hirajima, S.; et al. Circulating miR-18a in plasma contributes to cancer detection and monitoring in patients with gastric cancer. *Gastric cancer : official journal of the International Gastric Cancer Association and the Japanese Gastric Cancer Association* **2015**, *18*, 271-279, doi:10.1007/s10120-014-0363-1.
14. Zhao, G.; Jiang, T.; Liu, Y.; Huai, G.; Lan, C.; Li, G.; Jia, G.; Wang, K.; Yang, M. Droplet digital PCR-based circulating microRNA detection serve as a promising diagnostic method for gastric cancer. *BMC cancer* **2018**, *18*, 676, doi:10.1186/s12885-018-4601-5.
15. Emami, S.S.; Nekouian, R.; Akbari, A.; Faraji, A.; Abbasi, V.; Agah, S. Evaluation of circulating miR-21 and miR-222 as diagnostic biomarkers for gastric cancer. *Journal of cancer research and therapeutics* **2019**, *15*, 115-119, doi:10.4103/jcrt.JCRT_592_17.
16. Li, C.; Li, J.F.; Cai, Q.; Qiu, Q.Q.; Yan, M.; Liu, B.Y.; Zhu, Z.G. MiRNA-199a-3p: A potential circulating diagnostic biomarker for early gastric cancer. *Journal of surgical oncology* **2013**, *108*, 89-92, doi:10.1002/jso.23358.
17. Chen, X.; Cui, Y.; Xie, X.; Xing, Y.; Yuan, Z.; Wei, Y. Functional role of miR-27b in the development of gastric cancer. *Molecular medicine reports* **2018**, *17*, 5081-5087, doi:10.3892/mmr.2018.8538.
18. Parvaee, P.; Sarmadian, H.; Khansarinejad, B.; Amini, M.; Mondanizadeh, M. Plasma Level of MicroRNAs, MiR-107, MiR-194 and MiR-210 as Potential Biomarkers for Diagnosis Intestinal-Type Gastric Cancer in Human. *Asian Pacific journal of cancer prevention : APJCP* **2019**, *20*, 1421-1426, doi:10.31557/APJCP.2019.20.5.1421.
19. Hung, P.S.; Chen, C.Y.; Chen, W.T.; Kuo, C.Y.; Fang, W.L.; Huang, K.H.; Chiu, P.C.; Lo, S.S. miR-376c promotes carcinogenesis and serves as a plasma marker for gastric carcinoma. *PloS one* **2017**, *12*, e0177346, doi:10.1371/journal.pone.0177346.
20. Qiu, X.; Zhang, J.; Shi, W.; Liu, S.; Kang, M.; Chu, H.; Wu, D.; Tong, N.; Gong, W.; Tao, G.; et al. Circulating MicroRNA-26a in Plasma and Its Potential Diagnostic Value in Gastric Cancer. *PloS one* **2016**, *11*, e0151345, doi:10.1371/journal.pone.0151345.
21. Zhuang, K.; Han, K.; Tang, H.; Yin, X.; Zhang, J.; Zhang, X.; Zhang, L. Up-Regulation of Plasma miR-23b is Associated with Poor Prognosis of Gastric Cancer. *Medical science monitor : international medical journal of experimental and clinical research* **2016**, *22*, 356-361, doi:10.12659/msm.895428.
22. Hou, X.; Zhang, M.; Qiao, H. Diagnostic significance of miR-106a in gastric cancer. *International journal of clinical and experimental pathology* **2015**, *8*, 13096-13101.
23. Shin, V.Y.; Ng, E.K.; Chan, V.W.; Kwong, A.; Chu, K.M. A three-miRNA signature as promising non-invasive diagnostic marker for gastric cancer. *Molecular cancer* **2015**, *14*, 202, doi:10.1186/s12943-015-0473-3.
24. Fu, Z.; Qian, F.; Yang, X.; Jiang, H.; Chen, Y.; Liu, S. Circulating miR-222 in plasma and its potential diagnostic and prognostic value in gastric cancer. *Medical oncology* **2014**, *31*, 164, doi:10.1007/s12032-014-0164-8.
25. Zhu, C.; Ren, C.; Han, J.; Ding, Y.; Du, J.; Dai, N.; Dai, J.; Ma, H.; Hu, Z.; Shen, H.; et al. A five-microRNA panel in plasma was identified as potential biomarker for early detection of gastric cancer. *British journal of cancer* **2014**, *110*, 2291-2299, doi:10.1038/bjc.2014.119.
26. Cai, H.; Yuan, Y.; Hao, Y.F.; Guo, T.K.; Wei, X.; Zhang, Y.M. Plasma microRNAs serve as novel potential biomarkers for early detection of gastric cancer. *Medical oncology* **2013**, *30*, 452, doi:10.1007/s12032-012-0452-0.
27. Li, C.; Li, J.F.; Cai, Q.; Qiu, Q.Q.; Yan, M.; Liu, B.Y.; Zhu, Z.G. miRNA-199a-3p in plasma as a potential diagnostic biomarker for gastric cancer. *Annals of surgical oncology* **2013**, *20 Suppl 3*, S397-405, doi:10.1245/s10434-012-2600-3.
28. Li, B.S.; Zhao, Y.L.; Guo, G.; Li, W.; Zhu, E.D.; Luo, X.; Mao, X.H.; Zou, Q.M.; Yu, P.W.; Zuo, Q.F.; et al. Plasma microRNAs, miR-223, miR-21 and miR-218, as novel potential biomarkers for gastric cancer detection. *PloS one* **2012**, *7*, e41629, doi:10.1371/journal.pone.0041629.
29. Tsujiura, M.; Ichikawa, D.; Komatsu, S.; Shiozaki, A.; Takeshita, H.; Kosuga, T.; Konishi, H.; Morimura, R.; Deguchi, K.; Fujiwara, H.; et al. Circulating microRNAs in plasma of patients with gastric cancers. *British journal of cancer* **2010**, *102*, 1174-1179, doi:10.1038/sj.bjc.6605608.

30. Li, F.; Guo, Y.; Liu, J.; Zhang, R. The significance of elevated plasma expression of microRNA 106b~25 clusters in gastric cancer. *PloS one* **2017**, *12*, e0178427, doi:10.1371/journal.pone.0178427.
31. Zhou, H.; Guo, J.M.; Lou, Y.R.; Zhang, X.J.; Zhong, F.D.; Jiang, Z.; Cheng, J.; Xiao, B.X. Detection of circulating tumor cells in peripheral blood from patients with gastric cancer using microRNA as a marker. *Journal of molecular medicine* **2010**, *88*, 709-717, doi:10.1007/s00109-010-0617-2.
32. Valladares-Ayerbes, M.; Reboredo, M.; Medina-Villaamil, V.; Iglesias-Diaz, P.; Lorenzo-Patino, M.J.; Haz, M.; Santamarina, I.; Blanco, M.; Fernandez-Tajes, J.; Quindos, M.; et al. Circulating miR-200c as a diagnostic and prognostic biomarker for gastric cancer. *Journal of translational medicine* **2012**, *10*, 186, doi:10.1186/1479-5876-10-186.
33. Sharma, P.; Saraya, A.; Sharma, R. Serum-based six-miRNA signature as a potential marker for EC diagnosis: Comparison with TCGA miRNAseq dataset and identification of miRNA-mRNA target pairs by integrated analysis of TCGA miRNAseq and RNAseq datasets. *Asia-Pacific journal of clinical oncology* **2018**, *14*, e289-e301, doi:10.1111/ajco.12847.
34. Sharma, P.; Saraya, A.; Sharma, R. Potential diagnostic implications of miR-144 overexpression in human oesophageal cancer. *The Indian journal of medical research* **2016**, *143*, S91-S103, doi:10.4103/0971-5916.191796.
35. Bus, P.; Kestens, C.; Ten Kate, F.J.; Peters, W.; Drenth, J.P.; Roodhart, J.M.; Siersema, P.D.; van Baal, J.W. Profiling of circulating microRNAs in patients with Barrett's esophagus and esophageal adenocarcinoma. *Journal of gastroenterology* **2016**, *51*, 560-570, doi:10.1007/s00535-015-1133-5.
36. Bai, Y.; Lin, H.; Fang, Z.; Luo, Q.; Fang, Y.; Su, Y.; Hu, Q.; Duan, H.; Chen, F.; Zhang, Z.Y. Plasma microRNA-19a as a potential biomarker for esophageal squamous cell carcinoma diagnosis and prognosis. *Biomarkers in medicine* **2017**, *11*, 431-441, doi:10.2217/bmm-2016-0286.
37. Yao, L.H.; Wang, G.R.; Cai, Y.; Ma, Q.; Wang, D.S.; Xu, L.; Guo, X.L. [The expressions and diagnostic values of miR-18a and miR-21 in esophageal cancer]. *Zhonghua zhong liu za zhi [Chinese journal of oncology]* **2019**, *41*, 107-111, doi:10.3760/cma.j.issn.0253-3766.2019.02.006.
38. Shen, Y.; Ding, Y.; Ma, Q.; Zhao, L.; Guo, X.; Shao, Y.; Niu, C.; He, Y.; Zhang, F.; Zheng, D.; et al. Identification of Novel Circulating miRNA Biomarkers for the Diagnosis of Esophageal Squamous Cell Carcinoma and Squamous Dysplasia. *Cancer epidemiology, biomarkers & prevention : a publication of the American Association for Cancer Research, cosponsored by the American Society of Preventive Oncology* **2019**, *28*, 1212-1220, doi:10.1158/1055-9965.EPI-18-1199.
39. Zhang, H.; Shi, X.; Chang, W.; Li, Y.; Wang, L.; Wang, L. Epigenetic alterations of the Igf2 promoter and the effect of miR4835p on its target gene expression in esophageal squamous cell carcinoma. *Molecular medicine reports* **2018**, *17*, 2251-2256, doi:10.3892/mmr.2017.8134.
40. Huang, Z.; Zhang, L.; Zhu, D.; Shan, X.; Zhou, X.; Qi, L.W.; Wu, L.; Zhu, J.; Cheng, W.; Zhang, H.; et al. A novel serum microRNA signature to screen esophageal squamous cell carcinoma. *Cancer medicine* **2017**, *6*, 109-119, doi:10.1002/cam4.973.
41. Gupta, A.; Quijano, E.; Liu, Y.; Bahal, R.; Scanlon, S.E.; Song, E.; Hsieh, W.C.; Braddock, D.E.; Ly, D.H.; Saltzman, W.M.; et al. Anti-tumor Activity of miniPEG-gamma-Modified PNAs to Inhibit MicroRNA-210 for Cancer Therapy. *Molecular therapy. Nucleic acids* **2017**, *9*, 111-119, doi:10.1016/j.omtn.2017.09.001.
42. Wang, C.; Guan, S.; Liu, F.; Chen, X.; Han, L.; Wang, D.; Nesa, E.U.; Wang, X.; Bao, C.; Wang, N.; et al. Prognostic and diagnostic potential of miR-146a in oesophageal squamous cell carcinoma. *British journal of cancer* **2016**, *114*, 290-297, doi:10.1038/bjc.2015.463.
43. Dong, W.; Li, B.; Wang, J.; Song, Y.; Zhang, Z.; Fu, C.; Zhang, P. Diagnostic and predictive significance of serum microRNA-7 in esophageal squamous cell carcinoma. *Oncology reports* **2016**, *35*, 1449-1456, doi:10.3892/or.2015.4499.
44. Xu, H.; Yao, Y.; Meng, F.; Qian, X.; Jiang, X.; Li, X.; Gao, Z.; Gao, L. Predictive Value of Serum miR-10b, miR-29c, and miR-205 as Promising Biomarkers in Esophageal Squamous Cell Carcinoma Screening. *Medicine* **2015**, *94*, e1558, doi:10.1097/MD.0000000000001558.
45. Wu, C.; Wang, C.; Guan, X.; Liu, Y.; Li, D.; Zhou, X.; Zhang, Y.; Chen, X.; Wang, J.; Zen, K.; et al. Diagnostic and prognostic implications of a serum miRNA panel in oesophageal squamous cell carcinoma. *PloS one* **2014**, *9*, e92292, doi:10.1371/journal.pone.0092292.
46. Wu, C.; Li, M.; Hu, C.; Duan, H. Clinical significance of serum miR-223, miR-25 and miR-375 in patients with esophageal squamous cell carcinoma. *Molecular biology reports* **2014**, *41*, 1257-1266, doi:10.1007/s11033-013-2970-z.
47. Zhang, C.; Wang, C.; Chen, X.; Yang, C.; Li, K.; Wang, J.; Dai, J.; Hu, Z.; Zhou, X.; Chen, L.; et al. Expression profile of microRNAs in serum: a fingerprint for esophageal squamous cell carcinoma. *Clinical chemistry* **2010**, *56*, 1871-1879, doi:10.1373/clinchem.2010.147553.
48. Komatsu, S.; Ichikawa, D.; Takeshita, H.; Tsujiura, M.; Morimura, R.; Nagata, H.; Kosuga, T.; Iitaka, D.; Konishi, H.; Shiozaki, A.; et al. Circulating microRNAs in plasma of patients with oesophageal squamous cell carcinoma. *British journal of cancer* **2011**, *105*, 104-111, doi:10.1038/bjc.2011.198.
49. Wang, W.T.; Guo, C.Q.; Cui, G.H.; Zhao, S. Correlation of plasma miR-21 and miR-93 with radiotherapy and chemotherapy efficacy and prognosis in patients with esophageal squamous cell carcinoma. *World journal of gastroenterology* **2019**, *25*, 5604-5618, doi:10.3748/wjg.v25.i37.5604.
50. He, F.C.; Meng, W.W.; Qu, Y.H.; Zhou, M.X.; He, J.; Lv, P.; Ming, L. Expression of circulating microRNA-20a and let-7a in esophageal squamous cell carcinoma. *World journal of gastroenterology* **2015**, *21*, 4660-4665, doi:10.3748/wjg.v21.i15.4660.
